# Discovery and Validation of Novel Biomarkers for Detection of Epithelial Ovarian Cancer

**DOI:** 10.3390/cells8070713

**Published:** 2019-07-12

**Authors:** Hagen Kulbe, Raik Otto, Silvia Darb-Esfahani, Hedwig Lammert, Salem Abobaker, Gabriele Welsch, Radoslav Chekerov, Reinhold Schäfer, Duska Dragun, Michael Hummel, Ulf Leser, Jalid Sehouli, Elena Ioana Braicu

**Affiliations:** 1 Tumourbank Ovarian Cancer Network, 13353 Berlin, Germany; 2 Department of Gynaecology, European Competence Center for Ovarian Cancer, Campus Virchow Clinic, Charité—Universitätsmedizin Berlin, Corporate Member of Freie Universität Berlin, Humboldt-Universität zu Berlin, and Berlin Institute of Health, 13353 Berlin, Germany; 3 Institute for Computer Sciences, Humboldt-Universität zu Berlin, 12489 Berlin, Germany; 4 Insititute for Pathology, Charité—Universitätsmedizin Berlin, Corporate Member of Freie Universität Berlin, Humboldt-Universität zu Berlin, and Berlin Institute of Health, 10117 Berlin, Germany; 5 German Cancer Consortium (DKTK), Partner site Berlin, 10117 Berlin, Germany; 6 Department of Nephrology and Transplantation, Charité—Universitätsmedizin Berlin, Corporate Member of Freie Universität Berlin, Humboldt-Universität zu Berlin, and Berlin Institute of Health, 13353 Berlin, Germany

**Keywords:** biomarker discovery, ovarian cancer, tumour microenvironment, differential expression

## Abstract

Detection of epithelial ovarian cancer (EOC) poses a critical medical challenge. However, novel biomarkers for diagnosis remain to be discovered. Therefore, innovative approaches are of the utmost importance for patient outcome. Here, we present a concept for blood-based biomarker discovery, investigating both epithelial and specifically stromal compartments, which have been neglected in search for novel candidates. We queried gene expression profiles of EOC including microdissected epithelium and adjacent stroma from benign and malignant tumours. Genes significantly differentially expressed within either the epithelial or the stromal compartments were retrieved. The expression of genes whose products are secreted yet absent in the blood of healthy donors were validated in tissue and blood from patients with pelvic mass by NanoString analysis. Results were confirmed by the comprehensive gene expression database, CSIOVDB (Ovarian cancer database of Cancer Science Institute Singapore). The top 25% of candidate genes were explored for their biomarker potential, and twelve were able to discriminate between benign and malignant tumours on transcript levels (*p* < 0.05). Among them T-cell differentiation protein myelin and lymphocyte (MAL), aurora kinase A (AURKA), stroma-derived candidates versican (VCAN), and syndecan-3 (SDC), which performed significantly better than the recently reported biomarker fibroblast growth factor 18 (FGF18) to discern malignant from benign conditions. Furthermore, elevated MAL and AURKA expression levels correlated significantly with a poor prognosis. We identified promising novel candidates and found the stroma of EOC to be a suitable compartment for biomarker discovery.

## 1. Introduction

Epithelial ovarian cancer (EOC) is the fifth most common cause of cancer death in women in developed countries [1]. EOC is commonly referred to as the ‘silent killer’ due to a lack of specific symptoms that commonly leads to a diagnosis at a late and advanced stage with a 5 year survival rate of less than 40%. This 5 year survival rate, however, increases to over 90% when EOC is diagnosed at an early stage [2]. Hence, novel approaches to detect EOC earlier have great potential to achieve a meaningful impact on patient survival.

It is now known that ovarian cancer is a very heterogenous disease, with the major histological subtypes, serous, clear cell, endometriod, and mucinous, characterised by different somatic alterations and clinical etiologies. Since most identified tissue-based biomarkers differ significantly between subtypes [3], histology is important and has implications for biomarker studies. Nevertheless, even though the source of cell-of-origin and underlying biological mechanisms might differ considerably between the subtypes, common features, e.g., changes of the tumour microenvironment of the peritoneum in response to malignant transformation, are shared, and the most recently identified markers of the chloride intracellular channel (CLIC) protein family, CLIC1 and CLIC4, showed promising potential as serum and tissue biomarkers across all subtypes of EOC [4]. Biomarkers include gene expression products, metabolites, circulating nucleic acids characterised by somatic mutations, and splice variants [5,6,7,8,9,10]. The potential of microRNA signatures in the diagnosis and prognosis of ovarian cancer has been especially described in recent years [11,12,13,14]. Nevertheless, cancer antigen 125 (CA125) remains the most frequently used blood-based biomarker for ovarian cancer. However, CA125 can also be elevated in common benign gynaecological conditions, such as endometriosis, follicular cysts, and cystadenomas, especially in premenopausal women. Therefore, CA125 lacks the specificity to predict ovarian cancer. Moreover, CA125 is limited in the detection of early stage ovarian cancer, and, in fact, 20% of patients with advanced disease are CA125 negative [15]. Despite its limitations as a biomarker, CA125 has shown good specificity in combination with an ultrasound scan in postmenopausal women compared with a single serum test of CA125 alone. Jacobs et al. first described this algorithm called the risk of malignancy index (RMI) in 1990 [16]. However, both benign tumours and ovarian malignancies appear in ultrasounds as cystic or solid lesions. The presence of solid papillations will increase the risk of ovarian cancer from 0.6% in simple cysts to 33% in the presence of a papillation, according to the International Tumor Analysis Association (IOTA) criteria. Nevertheless, an ultrasound remains subjective and has to be performed by experienced sonographers. Even more important is the identification of highly suspicious lesions, which should be treated only in centres with high numbers of operations for optimal tumour debulking. Optimal debulking, as indicated by the lack of macroscopically residual lesions, is one of the most important prognostic factors [17]. It is more likely to be achieved in centres with a high turnover of surgical procedures performed by experienced gynaecological oncologists. To increase the diagnostic power of these parameters, human epididymis protein-4 (HE4), another common used biomarker in the serum of ovarian cancer patients, was used in a newly developed risk of ovarian malignancy algorithm (ROMA) especially to distinguish patients at a low and high risk of EOC [18].

HE4 detection shows less sensitivity compared with CA125 but exhibits higher specificity for malignant, rather than benign, conditions and may, therefore, increase diagnostic accuracy along with CA125 levels and ultrasound scans, respectively [19]. Indeed, significantly more low-volume cases of stage I and II EOC were identified in a high-risk population of germline mutation carriers in DNA repair associated breast cancer genes (BRCA) in a recent screening trial [20]. However, current screening strategies using CA125 velocity did not contribute to the overall reduction of mortality rates as shown in the UK Collaborative Trial of Ovarian Cancer Screening (UKTOCS) program [21]. Hence, the discovery of biomarkers, which improve the detection of EOC, is urgently required. 

Despite the heterogeneous nature of publicly available gene expression datasets, these have been instrumental in dissecting the underlying biological processes and pathways of tumor progression and in the discovery of biomarkers [22,23,24]. However, inconsistent findings in the biological and clinical characteristics of molecular signatures often occur as a result of the application of diverse statistical methods and transcriptional profiling on different platforms [23,25]. Another pitfall is that they are often limited regarding clinical variables and specific clinical outcomes within available datasets, preventing them from reaching their full potential to identify prognostic biomarkers or classifiers for clinical management. Moreover, the products of overexpressed genes have to be secreted to be traceable in the bloodstream. Vathipadiekal et al. established a library of secreted genes from publicly available databases, which allows identification of overexpressed genes encoding secreted proteins [22]. These can serve as blood-based biomarkers or indicate the absence of markers, e.g., in blood. The library has allowed researchers to validate known biomarkers and identify novel candidates such as fibroblast growth factor 18 (FGF18) and G-protein-coupled receptor GPR174A (GPR174A) [22].

In the past, tumour biology predominantly focused on epithelial tumour components as sources of biomarkers. Nevertheless, there is a complex mixture of malignant and non-malignant cells in multiple peritoneal tumour deposits, which show a dynamic network of paracrine interactions. It has been reported that the interplay between the network components impacts the overall progression or regression of the tumour, illustrated by the observation that advanced stages of EOC show a high stromal to epithelial ratio, which in turn is associated with a poor prognosis [26]. The stroma-epithelial interactions include soluble factors like chemokines, growth factors, and inflammatory cytokines, often as a consequence of oncogenic mutations within malignant cells [27,28,29,30]. The release of these factors into the blood has great potential as a biomarker. In general, the stromal compartment has been insufficiently considered as the origin of novel markers and needs further exploration.

A robust validation of novel biomarkers is extremely resource and time intensive and, thus, only the biomarkers with strongest prospects of efficacy can be validated. In this study, we have established a concept for blood-based biomarker discovery to detect ovarian cancer and extend the analysis by exploring the tumour’s microenvironment. We have used gene expression data obtained from the analysis of microdissected epithelium and adjacent stroma from benign and malignant serous tumours (GSE29156) [31], normal epithelium samples (GSE14407) [32], and microdissected cancer stroma (GSE40595) [33]. We retrieved biomarkers, including both stromal and epithelial specific candidates, and surveyed a panel of them in the tissue and blood of ovarian cancer patients for their potential as biomarkers. This method attempts to falsify biomarkers, i.e., identify false positive predictions with little potential of success. Predictions based on a small number of either transcripts or proteins in the blood have the potential to be more accurate to predict EOC in clinical management than methods solely based on CA125, HE4 levels, and ultrasound screenings.

## 2. Materials and Methods

### 2.1. Microarray Data and In Silico Analysis

We procured mRNA microarray data sets GSE29156, GSE40595, and GSE14407 from Gene Expression Omnibus (GEO) database [31,32,33,34]. GSE29156 describes microdissected epithelium and adjacent stroma from 23 benign and 27 malignant serous tumours, whereas GSE14407 contains expression data for 12 human ovarian cancer samples and 12 normal epithelia samples. Furthermore, we included the microarray dataset GSE40595, of which we analysed eight normal and 31 cancer stroma samples by conducting a differential expression analysis between these two sub-cohorts. All datasets underwent an initial quality control test based on the R-package ‘arrayQualityMetrics’, version 3.24.0 (doi.org/10.1093/bioinformatics/btn647), and we only utilised samples that passed the quality thresholds. We normalised probe set expression using the Affymetrix package’s RMA method (10.1093/bioinformatics/btg405). For GSE29156 and GSE14407, differential gene expression was calculated between benign and malignant epithelial and stromal tissue, respectively, by application of an empirical Bayes t-test using the R-package ‘Limma’ (https://doi.org/10.1093/nar/gkv007). Malignant stromal tissue expression was compared with benign stromal expression and malignant epithelial expression with benign epithelial expression. In the GSE40595 dataset, the differential expression was measured between normal and cancerous samples. *P*-values were adjusted for multiple testing using the Benjamini–Hochberg method [35]. Differentially expressed probes were selected on the basis of meeting the criteria of false discovery rate (FDR) *p* < 0.01. Probe sets that exhibited an absolute fold change of greater than two were utilised to generate heatmaps. Two-dimensional hierarchical clustering via differentially expressed genes, as well as principal component analysis (PCA) plots, were produced by the R-package ‘ggplot2′ [36] and heatmap. NanoString data were analysed using the R-package ‘NanoStringNorm’ [37] and also utilised to create the volcano plots.

### 2.2. RNA Extraction and Gene Expression Analysis

Whole blood samples from patients with either benign (*N* = 10) or malignant (*N* = 10) ovarian tumours were collected using BD PAXgene^TM^ Blood RNA System (2.5 mL; Qiagen, Hilden, Germany). Tubes were gently inverted 5 times directly after collection and incubated for 2 h at room temperature (RT) and then stored at −80 °C until processing. Samples were thawed on ice and left another 2 h at RT before RNA extraction using the PAXgene RNA extraction kit. All NanoString extractions were performed according to the manufacturer’s protocol. RNA from frozen tissue was extracted using Tri Reagent (Sigma, Steinheim am Albuch, Germany), and treated with 10 U DNase (Thermo Fisher Scientific, Schwerte, Germany). The malignant tumour cell content was assessed by an experienced clinical gynaecologic pathologist and was between 70–100% in the biopsies of ovarian cancer patients. Gene expression analysis was performed using the NanoString nCounter Analysis System (NanoString Technologies, Seattle, WA, USA) with a custom designed codeset containing 48 genes (Supplementary Table S1). Each reaction contained 250 ng of total RNA in a 5 μL aliquot, plus reporter and capture probes. Analysis and normalization of the raw NanoString data was performed using nSolver Analysis Software v1.1 (NanoString Technologies). Raw counts were normalised to the internal expression levels of beta2-microglobulin (B2M) and glyceraldehyde-3-phosphate dehydrogenase (GAPDH). We trained univariate and multivariate logistic regression models utilizing the standard R stats libraries and employed a logit-link function. The model was trained on tissue and blood samples that were housekeeping gene normalised.

### 2.3. Patients Characteristics

All samples used for the PAX gene and gene expression analysis were collected before surgery and after patients gave their informed consent at the Charité, Department for Gynaecology. The clinical study was approved by the Local Ethics Committee (EA2/049/13) and conducted according to the declaration of Helsinki. The serum samples used for enzyme-linked immunosorbent assay (ELISA) analysis were obtained from the BERLINER study, a prospective, multicentric study, that evaluated the additive value of HE4 and CA125 to ultrasound, to improve sensitivity and specificity, for the prediction of ovarian cancer in the pelvic mass patients. For this particular analysis, we only analysed the samples collected preoperatively at the Department of Gynaecology, Charité Medical University, Berlin, between 07/2013 and 10/2015, within the discovery cohort.

### 2.4. Circulatory Levels of Biomarkers by ELISA

Serum CA125 and HE4 protein levels were measured by the standardised method using the Roche ELecsys Platform at the core facility at Labor Berlin, Charité Medical University Berlin. FGF18 was quantified using commercially available ELISA kits from MyBiocource (Cat. no. MBS912811). All assays were performed following the manufacturer’s instructions. Protein levels were measured in duplicate and the mean values were used for statistical analysis.

### 2.5. Statistical Analysis

Statistical analysis of in vitro experiments used an unpaired *t*-test with Welch correction (GraphPad Prism version 4 Software, Software Inc., San Diego, CA, USA).

## 3. Results

### 3.1. Overview Biomarker Identification Concept

**Figure 1:**
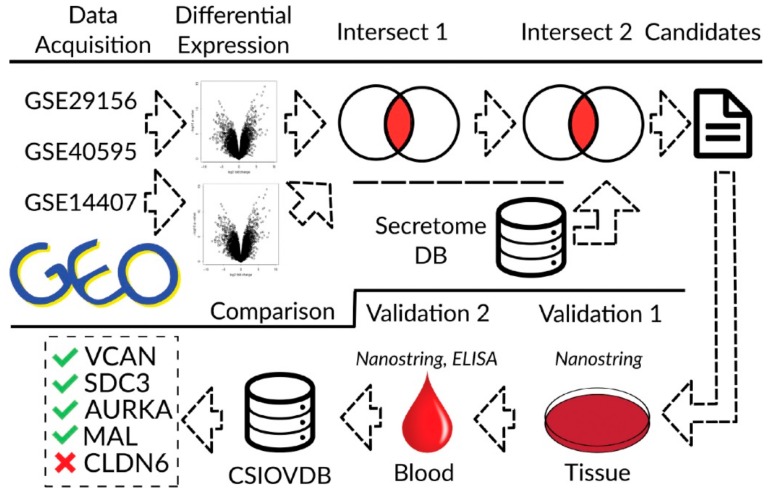
Overview of the biomarker identification concept. Three independent studies for genes over-expressed in malignant tissue were interrogated (Gene Expression Omnibus (GEO) series GSE29156, GSE40595 and GSE14407). Genes found to be over-expressed in both studies while simultaneously being secreted into the bloodstream were defined as biomarker candidates using the secretome database (DB). The candidates’ expression signatures in tissue and blood were measured by NanoString analysis and enzyme-linked immunosorbent assay (ELISA), respectively and compared to the reported signatures in the CSIOVDB database (Ovarian cancer database of Cancer Science Institute Singapore) to determine whether the measured signatures could be independently replicated. Versican (VCAN), syndecan-3 (SDC3), aurora kinase A (AURKA) and T-cell differentiation protein myelin and lymphocyte (MAL) were confirmed as potential biomarkers, but not claudin-6 (CLDN6) by this analysis.

**Figure 2:**
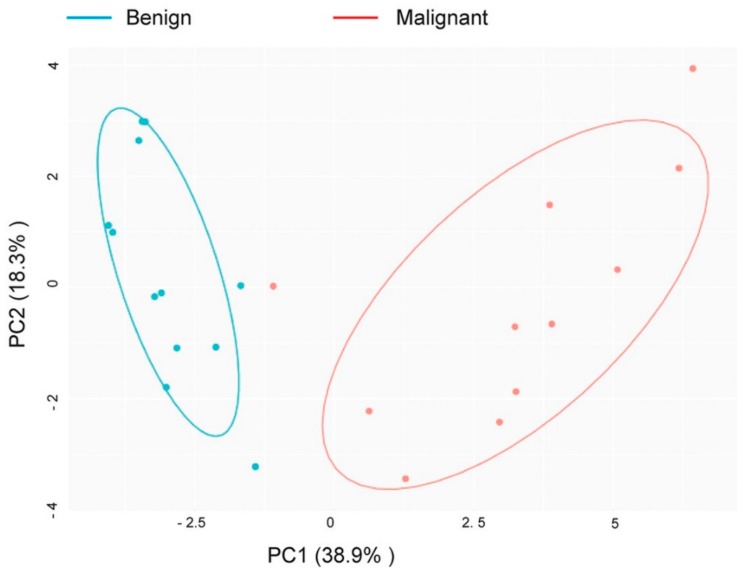
Principal component analysis (PCA) of patient-derived malignant and benign samples. Data from malignant and benign samples supported the pathological sample classification as malignant or benign since the sample were separable along the principal component 1 (PC1) of a principal component analysis (PCA) of their pairwise correlation. Their separability allowed identification of differentially expressed biomarker candidates to distinguish between benign and malignant samples.

**Figure 3:**
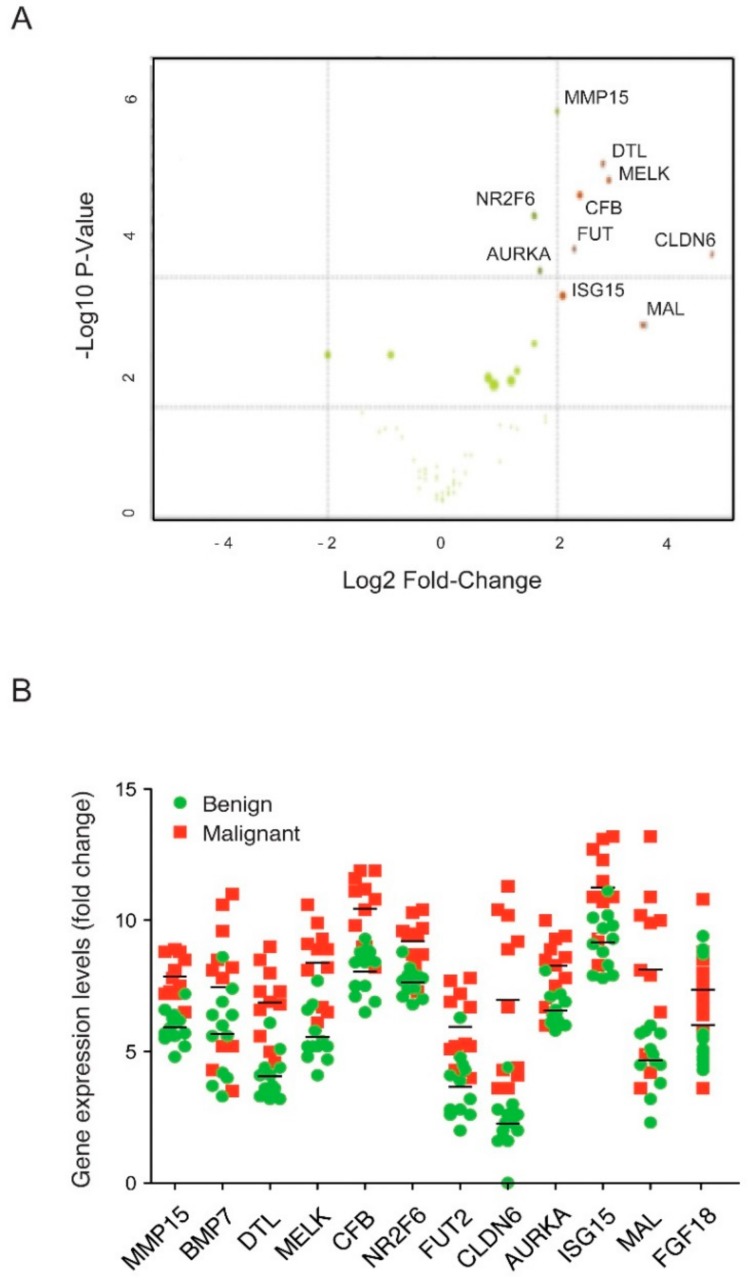
Validation of biomarker candidates in tissue and blood. (**A**) This plot depicts the Log–Fold changes and *P*-values of differential biomarker expression values between malignant (positive values) and benign tissue (negative values). The top 10 significant (*P*-value significance ≥ ~1.3) candidate biomarkers are labelled. (**B**) The distribution of gene expression levels of biomarker candidates matrix metalloproteinase 15 (MMP15), bone morphogenetic protein 7 (BMP7), denticleless E3 ubiquitin protein ligase (DTL), maternal embryonic leucine zipper kinase (MELK), complement factor B (CFB), nuclear orphan receptor (NR2F6), galactoside 2-alpha-L-fucosyltransferase-2 (FUT2), claudin-6 (CLDN6), aurora kinase A (AURKA), interferon-stimulated gene 15 (ISG15), myelin and lymphocyte protein (MAL), fibroblast growth factor 18 (FGF18) in benign and ovarian cancer tissues are shown (*p* < 0.05). The expression data were obtained by NanoString analysis using the mRNA from tissue samples of patients with benign (*N* = 10) disease or ovarian cancer (*N* = 10).

**Figure 4:**
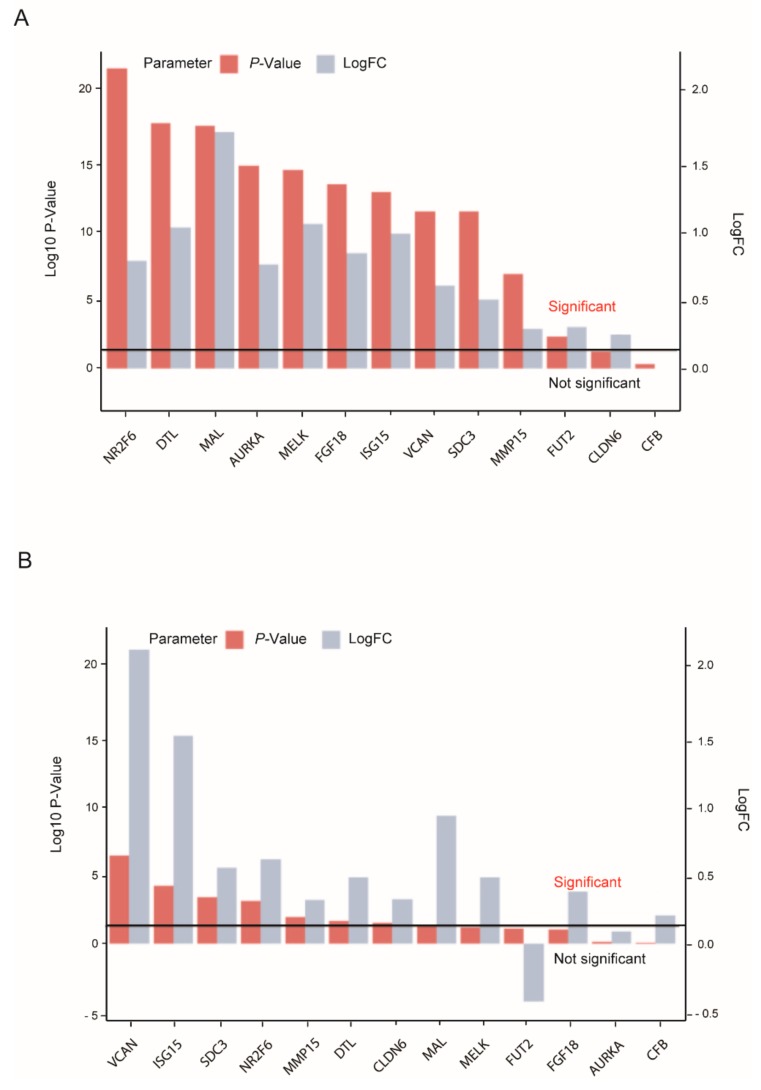
Reported biomarker expression. Log–Fold change of biomarker candidates are shown for two sets of cohorts; (**A**) healthy ovarian surface epithelium (OSE) versus ovarian cancer (OVCA) and (**B**) healthy versus cancerous stromal tissue. *P*-values higher than 1.3 are significant (horizontal line). Genes have been ranked according to their *P*-values in the OSE versus OVCA comparison from highest to lowest statistical power. Data have been procured from the CSIOVDB database [39]. Both plots show the same genes but are differently ordered by increasing the *P*-value. Differentially expressed biomarker candidates that distinguish malignant from healthy tissues are clearly present in plot A. By comparison, significantly fewer biomarkers that distinguish malignant from benign tissue are identifiable on plot B. In particular, the *P*-values for differential expression are significantly higher on plot B, although VCAN, ISG15, and MAL show a comparable Log–Fold change, which indicates a higher variance, i.e., expression heterogeneity within the groups.

**Figure 5:**
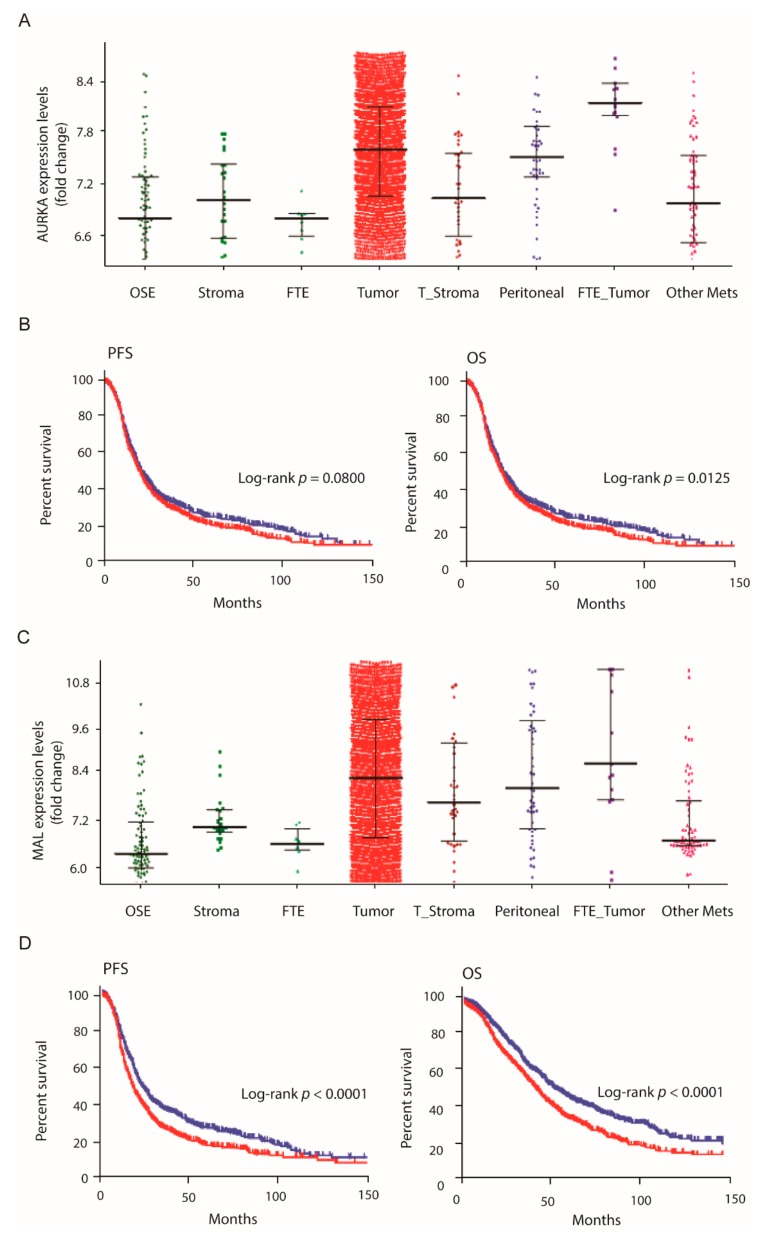
Gene-expression in ovarian cancer. Gene expression profiles of (**A**) AURKA and (**C**) MAL in normal tissue, including ovarian surface epithelium (OSE), stroma and fallopian tube epithelium (FTE), and the ovarian cancer disease state are shown. The correlation of gene expression with the PFS and OS of ovarian cancer patients is presented in (**B**,**D**), respectively. Kaplan–Meier plots were generated with samples of low (blue) and high (red) gene expression levels within the CSIOVDB dataset.

The concept to identify robust stromal biomarkers for the prediction of malignant EOC is shown in Figure 1. We identified genes differentially overexpressed in both malignant epithelium and stroma compared with benign control tissue from the GEO mRNA study, GSE29156. An independent gene expression dataset of ovarian cancer compared with the normal epithelium was included to expand the analysis. To assure that the differentially expressed genes encoded secreted proteins, we further restricted the list of candidates to those that were reported to be secreted by the secretome database of Vathipadiekal et al. The recently suggested biomarker FGF18 (3.6 fold) could be replicated. The biological processes that involve the biomarker candidates and distinguish malignant from benign EOCs were uncovered by a Gene Set over-representation analysis (GSOA) and could be classified as biologically plausible (Supplementary Figure S1). The top 25% of the Log-FC sorted 152 biomarkers candidates were validated on transcript levels in patient-derived tissue and serum with either malignant or benign tumours by NanoString analysis (Figure 2 and Figure 3, Supplementary Figures S2 and S3). Finally, the measured validation expression levels were compared to those entered in the independent CSIOVDB database (Ovarian cancer database of Cancer Science Institute Singapore) for confirmation (Figure 4 and Figure 5).

### 3.2. Detailed Description of the Identification Workflow

We pre-selected candidate biomarkers by analysis of published datasets and chose the mRNA-array GEO dataset GSE29156 [18] due to its focus on stromal tissue. Expression data from samples that were classified as healthy, benign, and malignant were available, with measurements of both the stromal compartment and epithelium. We compared (I) epithelium, (II) adjacent stroma, and (III) the complete transcriptome of 23 benign and 27 malignant samples and created lists of differentially expressed biomarker candidates provided in Supplementary Table S2.

Early detection biomarkers have to be both lowly expressed under normal and benign conditions and specifically elevated in the blood of ovarian cancer patients. To ascertain if candidate biomarkers fulfilled these conditions, we utilised a novel virtual secretome array consisting of 16,521 Affymetrix probe sets. We used this secretome array as the search space for potential candidates, using a more stringent cut off value of significantly overexpressed genes associated with malignant tumours (twofold; *p* < 0.01) to reduce the false positive rate. The lists of differentially expressed genes for the compartment specific signatures included 170 (I) (cut off twofold; *p* < 0.01) including 74 higher expressed in malignant epithelium and 193 genes (II) with 58 of them higher in the stroma of malignant biopsies, respectively. In our initial analysis, we found a unique set of 30 proteins within the secretome using either of the compartment specific gene lists. Using the total dataset with stromal and epithelial data combined to compare benign with malignant serous tumours, we identified 831 differentially expressed genes (III), with 268 of them higher in the ovarian cancer samples. A further 122 genes were detected when the more comprehensive gene list of genes was used in the same manner. Taken together, 152 genes were identified to be overexpressed in the mRNA expression dataset of malignant tumours compared with benign tumours, which could be potentially secreted into the bloodstream (Supplementary Table S3). Moreover, we included the gene expression data set GSE40595 [33], which contains microdissected normal and cancer stroma samples in the analysis, and found that 6103 genes were differentially expressed with higher expression levels in the malignant stroma compartment compared with normal controls (twofold; *p* < 0.01), with 81 (53%) of them also within the 152 gene signature.

A GSOA of the 152 secretome gene signature revealed the signature’s significant association with the processes and pathways found in ovarian cancer (Supplementary Figure S1). The expression levels of genes involved in extracellular matrix remodeling and integrin binding showed the highest overrepresentation in the malignant cases. Furthermore, we found increased levels of gene expression for members in the FGF, PI3K, and PDGF signalling pathways and downstream-regulated transcription factor (TF) signalling of activator protein 1 (AP1) and nuclear factor-κB (NF-κB) in biopsies of ovarian cancer compared with benign tumours. Other identified genes enriched in ovarian cancer belonged to the calcium regulation of molecular function categories, cholesterol metabolism, and the oxidative stress pathway.

Subsequently, the GSE14407 [32] mRNA dataset, comprising 12 normal and 12 ovarian cancer samples, was analysed to independently support or refute the candidate biomarkers. Between the groups, 3503 genes were differentially expressed (twofold; *p* < 0.01); 446 of them were higher in the ovarian cancer samples. The expression data were filtered for genes in the list of 152 biomarker candidates from the first gene expression analysis. 16 of them were identified to be more highly expressed in both the GSE40595 and GSE29156 datasets associated with ovarian cancer and found in the secretome array. Among them were nuclear orphan receptor NR2F6 (NR2F6), denticleless E3 ubiquitin protein ligase (DTL), myelin and lymphocyte protein (MAL), aurora kinase A (AURKA), fibroblast growth factor 18 (FGF18), maternal embryonic leucine zipper kinase (MELK), syndecan-3 (SDC3), and versican (VCAN).

### 3.3. Assessment of Biomarker Candidates in Tissue and Blood

To verify our findings we determined the mRNA expression levels of the top 25% (*N* = 38 genes) of 152 biomarker candidates in benign (*N* = 10) and malignant tumour (*N* = 10) biopsies by NanoString analysis. A further 10 genes were included for gene expression analysis according to the findings in the second dataset (GSE14407) associated with ovarian cancer and present in the list of 152 biomarker candidates within the secretome array. 56% of this list of 48 genes were also significantly overexpressed in the cancer stroma within the GSE40595 data set.

A principal component analysis (PCA) (Figure 2) of the pair-wise sample correlations supported the pathological classifications of the samples and the existence of differentially expressed genes, (Supplementary Figure S2). A PCA using the 48 gene signature showed a strong classification of benign and malignant samples, with the exception of one sample derived from a patient with low grade serous ovarian cancer (LGSOC), which presented a greater similarity with benign gene expression profiles. Genes differentially expressed between the shown cohorts were identified by a differential expression analysis (Figure 3A,B).

We expanded our study to include the gene expression profiles of the same panel of genes using mRNA samples from the blood of patients with benign conditions and ovarian cancer. No candidate biomarker was significant for differential expression. However, VCAN and SDC3 transcripts were elevated in the blood of ovarian cancer patients and showed a considerable trend toward significance (Supplementary Figure S3, *P*-values: VCAN = 0.052, SDC3 = 0.055).

We next estimated the predictive performance of the twelve genes whose expression was quantified in the tissue, as well as the performance of VCAN, shown in Supplementary Material Table S4. To that end, we trained a logistic regression model and predicted whether a sample was malignant or benign for each single gene by itself and once together, based on all twelve blood quantified genes. We observed that the predictive power in the tissue-derived NanoString quantified data was high, with an average sensitivity, specificity, and positive predictive value (PPV) of 88%, while the full model achieved a perfect predictive performance based on the tissue-derived data. The models trained on blood-derived data showed a reduced predictive power, with an average sensitivity of 68%, a specificity of 58%, and a PPV of 66%, and the full model achieved a sensitivity of 90%, a specificity of 70%, and a PPV of 75%.

### 3.4. Validation of Biomarkers in Serum

Since a high signal-to-noise ratio would increase the specificity of a biomarker in the detection assay, we analysed the protein expression of candidates in normal adult tissue using the human Proteome Map [38]. In order to validate the most promising biomarkers, based on the results of the PCA and volcano plot combined with the normal distribution pattern (Supplementary Figure S4), we decided to test the protein expression levels of the recently reported novel biomarker FGF18 in serum from women with a pelvic mass who were scheduled to have surgery.

The preoperative serum of patients with either ovarian cancer (*N* = 60) or common benign gynaecological conditions (*N* = 56) was included in this study. A detailed description of the patient cohort is shown in Table 1. First, we measured the most widely used tumour markers, CA125 and HE4, to compare against the newly suggested biomarker, FGF18, in terms of sensitivity and specificity. Both CA125 and HE4 levels were significantly elevated (*p* < 0.0001) in the serum of ovarian cancer patients in our study cohort (Supplementary Figure S5A,B). In the same samples, FGF18 protein expression levels were either not detected or were not significantly increased compared to serum from patients with benign neoplasia (*p* = 0.43) (Supplementary Figure S5C). However, FGF18 levels raised according to tumour stage (*p* = 0.0054) but were not correlated with any other clinical parameters like pre- and postmenopausal status, age, or tumour burden (data not shown).

### 3.5. Exploration of Potential Diagnostic Markers Using a Gene Expression Database

We queried the gene expression database CSIOVDB for whether the measured expression levels could be replicated independently [39]. The expression of selected biomarker candidates in healthy tissue (Figure 4A) was compared, as well as expression in ovarian cancer stroma (Figure 4B).

In particular, SDC3 and VCAN could be successfully replicated and were significantly overexpressed in tumour stroma compared to healthy stroma. NR2F6, DTL, MAL, and MMP15 could also be successfully replicated. However, they showed significantly higher *P*-values and lower Log-FCs. Notably, FGF18 and AURKA were specifically overexpressed in the malignant epithelium.

Apart from VCAN, SDC3, and MMP15, gene expression of these markers was increased with the grade and stage of the disease, regardless of the major subtypes significantly elevated in EOC (http://csibio.nus.edu.sg/CSIOVDB/CSIOVDB.html). On the other hand, genes like CLDN6 and CFB were not confirmed as potential biomarkers by this analysis.

Among all the investigated genes, AURKA and MAL expression levels showed the best correlation with progression free survival (PFS) and the overall survival (OS) of ovarian cancer patients, and high expression levels were associated with a poor prognosis. The correlation of AURKA and MAL expression levels with PFS and OS for patients with ovarian cancer is shown in Figure 5 as an example (Figure 5B,D). The Kaplan–Meier plots were obtained according to the low and high expression of AURKA and MAL and analysed using a log rank *P*-value calculated and displayed on the webpage.

Given the central role of the fallopian tube as a cell-of-origin source for a large proportion of high-grade serous ovarian cancer (HGSOC), we also compared the gene expression levels of candidates in the ovarian cancer epithelium with fallopian tube epithelium (FTE) expression. Only the stromal derived genes, VCAN and SDC3, as well as MMP15, were not found to be significantly overexpressed in this analysis, whereas all other candidates showed a strong significance (*p*-value <0.0001) and were associated with the malignant ovarian epithelium. Distribution in the gene expression of AURKA and MAL in FTE samples is shown in comparison with expression levels in other compartments in Figure 5A,C (Figure 5).

## 4. Discussion

Detection of EOC is urgently required to improve its prognosis and save the lives of women worldwide. The need persists for a differential diagnosis that distinguishes pelvic mass patients with either benign gynaecological conditions (including benign tumours and cysts) or EOC. CA125 is still the most common serum biomarker used clinically to detect EOC, despite its limitations, specifically due to fact that it is associated with the menstrual cycle and stages during pregnancy. It can also be overexpressed by inflammation and in common gynaecological conditions, such as endometriosis [40]. Therefore, biomarkers based on detecting ovarian cancer specifically in blood-based assays need to be validated in a clinical context with benign and malignant gynaecological conditions. Sensitivity is also an issue since not all ovarian cancer expresses CA125.

Many studies report the discovery of different potential biomarkers, but most of them do not meet the criteria of sensitivity and specificity [4,41,42,43,44,45]. However, no biomarkers outperformed CA125 [46,47]. One strategy to improve these parameters is to use the combinatorial power of different biomarkers. Especially in the light of inter- and intra-tumour heterogeneity, multiple tumour-specific molecules might be needed for detection. Further, the contribution of the tumour microenvironment in response to the process of malignant transformation and in tumour progression has often been neglected and needs further evaluation.

In addition, several of the proposed biomarkers have not been thoroughly validated, which entails a lack of information on the history of the samples, as well as standard operating procedures for sample selection, collection, and storage. Moreover, validation studies frequently compare healthy and diseased cohorts, which often do not match by age [2,12]. Therefore, in our study we used well controlled serum samples from patients with a pelvic mass (*N* = 56, benign gynaecological conditions and *N* = 60, ovarian cancer) undergoing a surgical procedure at the Department of Gynaecology at the Charité in Berlin. To be able to compare the performance of novel candidates with established markers CA125 and HE4 in predicting ovarian cancer, we first determined the levels of CA125 and HE4 in our cohorts.

Here, we queried publicly available gene-expression data of microdissected stroma and epithelial tissue to identify novel stroma-based biomarkers that discern ovarian cancer tissue from benign gynaecological conditions [18]. By filtering for genes differentially expressed not only between malignant and benign epithelial tissue but also between stromal tissue, we identified 152 novel biomarker candidates involved in the processes and pathways that differentiate ovarian cancer from benign gynaecological conditions.

Dysregulation and increased activity of the FGF, PDGF, and PI3K pathways were strongly associated with malignant tumours, and, therefore, a significant fraction of the biomarker candidates was associated with these processes. Increased activity of these pathways is supported by recent findings, e.g., with respect to PI3K, which is reported as being activated in up to 50% of high-grade serous ovarian cancers (HGSOCs) [5,22]. Furthermore, biomarker candidates show a high degree of connectivity due to their common involvement in the transcriptional regulation programs of AP1 and NFκB.

Since a significant fraction (53%) of the 152 gene signature were found also to be increased in malignant stroma compared with normal control samples, one can speculate that a substantial proportion of the biomarker candidates are part of a stroma response to cancer progression. This result shows once again the value of this compartment as a source for biomarker discovery.

Only biomarkers with the strongest prospect of efficacy can be validated in subsequent high-sample size studies due to cost and resources. Therefore, attempts to falsify biomarkers, i.e., identify false positive predictions with little potential of success, is imperative. To assess the real-world efficiency of the 152 biomarker candidate panel, we restricted this list by selecting 48 genes, which were simultaneously secreted according to a comprehensive secretome database, within the top 70th percentile of Log-FCs with the greatest potential [22].

Estimation of the false positive candidates was based on NanoString analysis and performed on tissue and the peripheral blood of patients with EOC and benign gynaecological conditions. 12 genes were significantly differentially expressed in tissue with an expression at least twice as high in malignant samples. The top genes were MMP15, DTL, MELK, CLDN6, AURKA, and MAL (Figure 3). FGF18 was also significantly overexpressed but to a lower degree. We did not find any of the stromal specific genes to be significantly differentially expressed within the tissue. This is possibly because the malignant cell content of >70% within the tissue samples induced a bias towards genes differentially expressed within the epithelium. In contrast, we did not find genes from the malignant epithelium, but transcript levels showed the stroma-derived genes, VCAN and SDC3, to be elevated in the blood of patients with EOC. Although the results obtained in blood fell short of significance by a narrow margin (*P*-value VCAN 0.052, SDC3 0.055, Supplementary Figure S3) the impact of the tumour microenvironment has to be taken into account since both genes are also expressed in other cell types, such as macrophages, endothelial cells, and fibroblasts [48,49,50]. 

Even though the utilised sample size of ten candidates for each condition is not sufficient for a clinical validation in the context of a false-positive biomarker candidate exclusion study, as described above, it can be considered sufficient. An optimal choice of technology, e.g., either miRNA or protein detection in conjunction with an increased sample size can be reasonably assumed to render the markers’ performance significant. However, the purpose of this study is to identify and exclude markers whose validation is not promising and to report marker candidates that show potential for being effective markers despite a limited sample size and unoptimised technology. Notably, VCAN and SDC3 overexpression signatures were independently replicated by querying the CSIOVDB. Stromal VCAN expression is induced by TGFβ and IL6 and has been shown to regulate processes like tumour growth and invasion. Furthermore, VCAN expression levels correlate with tumour progression and are a strong prognostic indicator, particularly in stage II colon cancer [51]. Therefore, VCAN has the potential to become a promising biomarker for ovarian cancer. Syndicans are another class of secreted extracellular matrix glycoproteins that have an important role in cancer development and prognostic value in various tumours, including ovarian cancer [52,53].

The protein levels of CA125 and HE4 within blood were significantly elevated in patients with ovarian cancer compared to benign conditions (Supplementary Figure S5A,B). However, FGF18 was not confirmed as a biomarker in our sample cohort. It is important to point out that the previous ELISA validation study by Vathipadiekal et al. was performed on a small number of serum samples from late stage III/IV patients compared with samples from normal probands [22]. Nevertheless, FGF18 protein levels correlated with disease status in our study and increased with tumour progression (Supplementary Figure S5C).

From the set of genes we found to be dysregulated and associated with malignant ovarian cancer, AURKA and MAL seemed to have the greatest impact on OS. Aberrant Aurora-A kinase activity has been generally implicated in oncogenic transformation and tumour progression [54]. Furthermore, it has not only been shown to be a therapeutic target in several different cancer types but to also have potential as a biomarker in colorectal, gastrointestinal, and bladder cancer [55,56,57]. MAL has been shown to regulate proliferation and mediate platinum resistance in EOC [58]. Overexpression of MAL is an independent predictor of poor survival and is, in particular, a feature of HGSOC and other subtypes of EOC [59,60]. Since AURKA and MAL have been implicated in ovarian cancer pathology and are, therefore, functionally important rather than the outcome of a deregulation side effect, they are promising nominees to include in a panel of novel biomarkers for the detection of ovarian cancer.

As a result, the measurement of two or more transcripts obtained by PAXgene tubes in blood has the potential to be more reliable and robust in the prediction of overall survival and merely requires a direct draw into the tubes to minimise RNA degradation at room temperature. This analytical validation of real-time reverse transcription polymerase chain reaction (RT-PCR) based assays to detect transcripts was performed using tissue and peripheral blood from metastatic prostate cancer patients [4]. However, further evaluation of the transcript levels in blood needs to be performed on a bigger cohort of patient samples. Since multiplex technologies such as NanoString gene expression assays can analyse a large number of different biomarkers in a single experiment, the study should be expanded to include the entire 152 gene signature we identified to be significantly overexpressed in malignant, compared with benign, tumours while being secreted. In this context, it is worth mentioning that there are many reasons why the results obtained from tumour tissue are different from the transcript levels in blood and are not detected there. One obvious answer is the issue of degradation from release until detection in a sensitive blood-based assay. Moreover, in contrast to their products, mRNA is not actively released or secreted. Thus, a direct correlation between blood-based assays and transcript levels might not always be possible. Therefore, it would be more relevant to assay the protein levels of the top candidates in blood, rather than RNA, but this is beyond the scope of the current study.

In summary, several markers showed substantially elevated expression levels between ovarian cancer compared with benign conditions in publically available expression data and our proof of concept study (including recently suggested marker FGF18) for ovarian cancer prediction [22]. Nevertheless, mRNA expression did not necessarily translate into protein levels (Supplementary Figure S5). Further candidates of novel biomarkers for validation by ELISA should be selected based on high values of gene expression and *P*-values in ovarian cancer tissue and blood by NanoString analysis (Figure 3, Supplementary Figures S2 and S3), as well as low or absent protein expression in a healthy cohort [38]. While MMP15 was still significantly overexpressed in ovarian cancer biopsies, CLDN6 did not meet these criteria in a bigger cohort (Figure 4A). In support of those findings, MMP7 has already been reported to be a suitable biomarker in EOC [44]. Moreover, NR2F6, DTL, MAL, and AURKA showed greater differential expression than MMP15 and FGF18 in the epithelium of EOCs and hence might have greater potential as biomarkers as their evaluation can prove advantageous.

It should be mentioned again that all utilized discovery gene expression datasets analysed in this study were generated using biopsies of HGSOC patients. However, it is now clear that the term ‘ovarian’ cancer refers to at least four distinct diseases, all of which grow and spread within the peritoneal cavity and ovary. However, by querying the extensive CSIOVDB database with microarray data from over 3000 EOC biopsies, including profiling the stromal compartments to analyse those independently, the identified candidates were significantly increased across all EOC subtypes and were, therefore, not only exclusively relevant to HGSOC.

Although the fallopian tube is thought to be the most common place of origin for ovarian cancer, OSE cannot be ignored, as not all HGSOC cases can be explained as an evolution from serous tubal intraepithelial carcinomas (STICs). Due to the lack of gene expression data for FTE in the discovery datasets, biomarker candidates were identified by comparing the gene expression profiles of ovarian cancer with microdissected normal and benign OSE and, for consistency, were presented using the comprehensive database CSIOVDB (Figure 4A). Since FTE data were available in this database, a comparison of cancer epithelium versus FTE was also included and, apart from the stromal derived candidates VCAN and SDC3, as well as MMP15, all other discovered biomarkers were associated with EOC and increased with tumour progression. Although there are no gene expression profiles available for benign tumours, it is worth noting that we do not aim to validate, but rather determine biomarker candidates with the greatest potential of success utilising the CSIOVDB database. These would then need to be validated in subsequent follow-up studies in the serum of patients with benign gyneacological conditions and EOC.

Further studies will focus on the evaluation of AURKA and MAL in combination with a panel of candidates with the substantial differential expression identified in this study. This will include genes such as VCAN and SDC3 from the stromal compartment for the detection of EOC in blood based assays.

## 5. Conclusions

The aim of the study was to render a differential diagnostic approach possible the discovery novel biomarker candidates across all major subtypes of EOC. We have established a concept for blood-based biomarker discovery to detect ovarian cancer and extended the analysis by exploring the tumour microenvironment, because the stroma represents a viable source of biomarkers, but often neglected. We retrieved novel biomarker candidates from public databases, including both stromal and epithelial specific. We provide evidence that the tumour stroma might be a useful source for biomarker discovery to predict EOC. The identified candidates should be included and subject of future biomarker research also for early detection of EOC.

## Figures and Tables

**Table 1:** Clinico-pathological parameters of the patient cohort.

Clinical Parameters	Tissue	Blood	Serum
Benign pelvic tumours			
Age at first diagnosis (median/range)	49 (25–68)	69 (41–92)	47 (23–79)
CA125 (U/mL) mean (range)	72 (12–278)	18 (6–77)	28 (5–215)
He4 (pM) mean (range)		44 (32–78)	52 (30–90)
Histology (*)			
Cystadenoma	3 (33%)	2 (20%)	19 (33.9%)
Dermoid cyst	3 (33%)	2 (20%)	12 (21.4%)
Endometriosis	2 (20%)	4 (40%)	8 (14.4%)
Functional cysts	2 (20%)	1 (10%)	4 (7.1%)
Myoma uteri	2 (20%)		1 (1.8%)
Benign Brenner tumour			1 (1.8%)
Cystadenofibroma			4 (7.1%)
Fibroma			2 (3.6%)
Others		2 (20%)	5 (8.9%)
Ascites			
Present	1 (10%)		3 (5.4%)
Absent	9 (90%)	10 (100%)	52 (92.9%)
NA			1 (1.7%)
*Ovarian Cancer*			
Age at first diagnosis (median/range)	61 (48–79)	58 (29–86)	62 (22–79)
CA125 (U/mL) mean (range)	1046 (12–6193)	600 (10–3331)	1124 (8–11616)
He4 (pM) mean (range)	341 (49–1305)	892 (97–3136)	637 (47–4676)
Histology			
High grade serous	6 (60%)	9 (90%)	46 (76.7%)
Low grade serous	1 (10%)		1 (1.7%)
Endometrioid	1 (10%)		9 (15.0%)
Mucinous	1 (10%)		1 (1.7%)
Clear cell	1 (10%)	1 (10%)	2 (3.3%)
Others			1 (1.7%)
Grading			
G1	3 (30%)		7 (11.7%)
G2–3	7 (70%)	10 (100%)	53 (89.3%)
FIGO Stage (**)			
I–II	2 (20%)		12 (20.0%)
III–IV	7 (70%)	10 (100%)	48 (80.0%)
NA	1 (10%)		
Ascites			
Present	6 (60%)	7 (70%)	32 (53.3%)
Absent	4 (40%)	3 (30%)	28 (46.7%)

* One patient had both uterus myomatosus and a functional cyst, one patient had endometriosis and cystadenoma, and another patient had both a dermoid cyst and cystadenoma. ** Fédération Internationale de Gynécologie et d’Obstétrique (FIGO).

## Supplementary Materials

The following are available online at https://www.mdpi.com/2073-4409/8/7/713/s1, Figure S1: Gene-set over-representation analysis of candidate biomarkers, Figure S2: Expression levels of candidate biomarkers and pair-wise correlation of underlying samples, Figure S3: Volcano plot of malignant versus benign gene expression in PAXgene samples from blood, Figure S4: Normal protein expression levels of biomarker candidates in tissue from healthy donors, Figure S5: Protein expression levels of biomarkers in serum, Table S1: List of custom probes designed by NanoString, Table S2: List of differentially expressed genes between benign and malignant epithelial and stromal tissue, Table S3: 152 gene signature, Table S4: Biomarker prediction analysis.
