# Supplementary material for: Discovery and Validation of Novel Biomarkers for Detection of Epithelial Ovarian Cancer
Source: Cells. 2019 Jul 12;8(7):713. doi: 10.3390/cells8070713 (PMC6678810; doi:10.3390/cells8070713)
Supplement: Supplementary file 1 [file cells-08-00713-s001.zip › Suppl. Table 1.pdf]

Table S1. List of custom probes designed by Nanostring

Gene	Target sequence
B2M	CACGGAGCGAGACATCTCGGCCCGAATGCTGTCAGCTTCCTCAAGACCTAAGCGACAGCGTGACCTTGTTTCA
EFEMP1	TGACTTCACGAGCACAAGCATTGCACTTACAGGACTTGTTTGTCGTAGGTCATCCTCTTCTTTTCTTGGTGTTGAGAAGATGCTC
SLIT2	AACTCTGTGACATCTCTTGGAATACCTTTCGGCAAGACCTTCAAACCCTTCACAATTCTGCGGGTTAGCAGGAAGGTTAGGGAAC
RGS1	TCTGTTAATTCCCTTCAGCCAGGGACCAGTCACTTTAGGCTATTAGCCTGCTGTTGAGATTATTGAGCTTCATCATGACCAGAAG
DTL	ACTTAGAAAAGGCAACTGACTTGAGGCTGCATTGATGACCTTTGCATGTTCAAAGACGCCTATCTTCCAGTTTGATCGGGAAACT
CD53	GGTTTTGCATTTCCGGAGATGAAACAAGTCTCTTCACCACAGGACTACTGCGAACCTAACTCCTCGCTACATTCCTATTGTTTTC
MMP15	ACAAAACGACCGTCTTGGCGCTCGTAGGCAGCACTGATGTCACCGGCCAATTTGGTTTTACTCCCCTCGATTATGCGGAGT
ICMT	AACCAAGCGTACACTCCACTGGTCACCAGAGTATGTGTATCTGATTTTTCCTTTCGGGTTATATCTATCATTTACTTGACACCCT
SPP1	CTGACTCGTTTCATAACTGTCCTTCCCACGGCTGTCCCAATCAGAAGGCGCAACAGCCACTTTTTTTCCAAATTTTGCAAGAGCC
AURKA	AAGGAAATTGCTGAGTCACGAGAACACGTTTTGGACCTCCAACTGGAGCTCACCGTGTGGACGGCAACTCAGAGATAACGCATAT
PLEK	GAGCTGCTGCTACTTGTGCTTAAAGCTGAGGCACGAACGCAATATCATTTCCTGGAGTTTATGTATTGCCAACGAGTTTGTCTTT
APOLD1	CCCTAATGGGCTTTTAGTGTTACATCCCTTGTTCATACGGTGAGACACTGCAGATAAGGTTGTTATTGTGGAGGATGTTACTACA
ITGA4	TCGCCCCGGGATTGATCACTGAAGCGTTGGCGAGCCAGTTGCTTCCTTCCTGTGTTCCAGCTACAAACTTAGAAAC
ISG15	CTCAGAGGTTCGTCGCATTTGTCCACCACCAGCAGGACCATAAAATTGGTTTTGCCTTTCAGCAATTCAACTT
GAPDH	AAGTGGTCGTTGAGGGCAATGCCAGCCCCAGCGTCAAAGCTGGTCAAGACTTGCATGAGGACCCGCAAATTCCT
CFB	CGGTGCAGGGGAGACAAATGGGCCTGATAGTCTGGCCATATTTCAGCTTACTTTCGTTGGGACGCTTGAAGCGCAAGTAGAAAAC
RAPH1	GATAATCCTTTCAAGGTGCCATGTTTCAATGTATGTCGGCTAACAGGCAACCAGCAGACCTGCAATATCAAAGTTATAAGCGCGT
S100A8	AATTTCTTCAGGTCATCCCTGTAGACGGCATGGAAATTCCCCTTTATCAGCCTGCCAATGCACTCGATCTTGTCATTTTTTTGCG
NANP	CCTGTGGGAGAGTTAAAAGGACAAGCCCCCTGCAGCACACGCCAACAAACTGGAGAGAGAAGTGAAGACGATTTAACCCA
OAS2	CTGGATGGTGAACCCATCAAGGGACTTCTGGATCTCGAAATTGTTTTTCACGATTGCTGCATTCCGCTCAACGCTTGAGGAAGTA
VCAN	CAAACATTTGTTCTTCGTGAGACAGGATGCTTGTGAGATGGGCACCCTGCCTGAGGCTGTTAAAGCTGTAGCAACTCTTCCACGA
CLDN6	CTCTTTGGCTCCATCATCCAAGGGTGAGAAACAGCAGAGCCTAAGCTAGGACGCAAATCACTTGAAGAAGTGAAAGCGAG
LRP2	AGCACCAAACCTAGAGCCCTCCCCTCGCACAGTGTAATACACAACCCACGCGATGACGTTCGTCAAGAGTCGCATAATCT
NPC2	GTTGGGTGGGGCATGGGCTCACATTCACTTCCTTTATAACTCCATCCACACATTTGGAATGATGTGTACTGGGAATAAGACGACG
RALBP1	TCATTTTAGAAGGACTTTCTGCCTCATCTCCAGAGCTATCTTCCTGAAAGCACAAGAATCCCTGCTAGCTGAAGGAGGGTCAAAC
NR2F6	TACGTGTCCAGAGGATCTGCCTGGCACACGCTAGCTACCTTGACGTAGATTGCTATCAGGTTACGATGACTGC
ARL4C	CCACACACACACACACCCCATCCAGCATTTACACCAAAAGCCTTACCCTTCTTACAGATCGTGTGCTCATGACTTCCACAGACGT
TINAGL1	ATCTCCTTGTCGTTGGAGCCGAGGCGGTAGACAGGCTTGGAGGAGTTGATAGTGGTAAAACAACATTAGC
LMF2	CCCACAAGGGACAGTTGGACTACAGCACTGAGCTTCCGTAGCCTACGTATATATCCAAGTGGTTATGTCCGACGGC
FGF9	CCCATCCAAGCCTCCATCATACATTATGCATGCGACAAAGTTTGGCAACACAGCAAGAAGGAGTATGGAACTTATAGCAAGAGAG
UTRN	AACCTGGAGGTTGGCATCATCCAGCTCAGATACTAAACGCTTCACAATTTCACCCCTCCAAACGCATTCTTATTGGCAAATGGAA
FGF18	ATCGGGCTTCCCCACGAGCTTGCCTTTGCGGTTCATGCACAGGTAGAATTCCCGAAGCAATACTGTCGTCACTCTGTATGTCCGT
BMP7	GTAGCGTGGGTGGAAGAATTCCTTGTCATGTTCCACGAGGTTGACGAAGCCCGGGAATCGGCATTTCGCATTCTTAGGATCTAAA
GPRC5B	AGTGGTTGATCACTTTCCAAATGTGATGGTCACTTCATCACCCAGAGGGCCCGATCTTCATAACGGACAAACTGAACGGGCCATT
THBD	TGGCACTGGTACTCGCAGTTGGCTCTGAAGCACGGCGCTATGCAGACGAGCTGGCAGAGGAGAGAAATCA
TNFSF10	CTGTGAAGATCACGATCAGCACGCAGGTCTGTCCCCATTCGCAACCATGTGAAGTAATGTGAGCGTACTT
FUT2	GAAGAGGCAGGACCTATGGACTTGGATGCATGCCAGCTACAAGCCAATATCACCAGTTAGCGTGGCGTATACCATGTTGTTAACA
CLIC4	TGAGAAAACCCTCACTCCCTCTAAACTTTTAGCTTCTACATGGTGTCAGACCTGAATCAATAGAACAATATCAGTTATGGCGGTG
SPRY1	TGAACTTGTGCTGTGTCAGGTCCTCTTTCAAGGAACCCTTCAAGTCATCCCGGTTGTTAATATGACAGGCCGCTAAAGACGTTCT
PRIMA1	TCCAGTACACGCGGCACGTTGCTAAGAAGGCAGATTTCAGGATATTCACACCGTCTCAGATGAGTGGGTTAATCAATCAAGTATG
CALR	AGGCCCAGCACGCCAAAGTTATCATAGGCATAGATACTGGGATCGGCTGACACATTAGTAACGTCGGCAAGCACTTAGTCG
SDC3	AGAGGAGAGATTCAACAACCCCAGAGAGACCAGGACAAAACAGGCTGGTGCGTGAACCAGATTATGTATGGACGCGCAATAGATA
SLC6A8	AGAAGGTTCCATTGCTGGCTGGCTCTCTAAGCTTCTATAACAGGGCATACGAAATTTGAGCAAGCAATTGAAGGCTTAGA
IFITM1	TCTGTAACATAATATGGTAGACTGTCACAGAGCCGAATACCAGTAACAGGCTATCAGCTAATAGGGTCGGCTCAACAGTGTATCC
ADAMTSL5	ATCAGGTAAAGAAGACAGGAGACGTGGAGCCCGGTATGGAGAGGAAGAACCTATCAATTCGTGACCCCGATCATCCAGTCCAGAA
MAL	ACAGCAGTTGGAGGTTAGACACAGCAAGCTCCCAACCCGGACGCGAAGCTTGAGCTCTAGGCCCAAAACGACCTTAATGGTCA
PTP4A2	ACTGAACACAGCAATGCCCATTGGTATCTCTGAAGCGTAATCGCATCTTACTAGCCCAGATCCTACGAGATGAGCTACGTAACTA
MELK	GAATGCTACTGGGAGAGAGCCACTTGGGAACATCATATTTTCCTCTCATACAAATGCACTCTATATGGAGGGAGAGTAGCTGGAT
