# Supplementary material for: Discovery and Validation of Novel Biomarkers for Detection of Epithelial Ovarian Cancer
Source: Cells. 2019 Jul 12;8(7):713. doi: 10.3390/cells8070713 (PMC6678810; doi:10.3390/cells8070713)
Supplement: Supplementary file 1 [file cells-08-00713-s001.zip › Suppl. Table 2.pdf]

Table S2. List of differentially expressed genes between benign and malignant epithelial and stromal tissue

(I) malignant versus benign epithelium

HGNC_symbol	logFC
CENPF	4,12
CDC6	3,43
CLSPN	3,17
RGS2	3,01
RRM2	2,95
DTL	2,92
DLGAP5	2,91
PRDM1	2,8
CENPF	2,77
MMP15	2,69
IQGAP3	2,67
ICMT	2,52
SPP1	2,5
AURKA	2,46
CENPF	2,35
WDR76	2,29
ISG15	2,2
SPP1	2,08
ICMT	2,04
ORC6	1,99
C6orf203	1,95
CLDN6	1,86
LRP2	1,86
NPC2	1,85
H1F0	1,83
RALBP1	1,81
CHCHD3	1,8
APEX2	1,8
C19orf55	1,79
C1orf106	1,76
SOX17	1,76
GPX1	1,69
CRABP2	1,69
IFI44L	1,61
NDC80	1,55
CALR	1,52
UBE2D3	1,51
NCOA7	1,51
GATAD2A	1,5
NOTCH3	1,47
PTP4A2	1,47
FGF9	1,46
MELK	1,45
RHBDL2	1,43
MIOS	1,41
ZFYVE16	1,41
TSEN54	1,39
PHOSPHO2-K	1,38
BHLHE40	1,36
KIAA1143	1,32
SNX5	1,27
LOC284023	1,26
PCBP1	1,26
ZNF770	1,24
MYH7	1,23
MRPS12	1,21
XPO4	1,2
SCD	1,19
RASGEF1B	1,19
LRRC37A2	1,18
VKORC1L1	1,17
FUCA2	1,16
KIAA1654	1,15
ZNF525	1,11
SEMA4C	1,09
AP2M1	1,07
ATP6V0A2	1,06
RPN2	1,06
PFDN2	1,05
RALY	1,05
S100A8	1,02
SCNM1	1,01
CYP4V2	1,01
SNRPG	1
CTBP1-AS1	-1
UBE2J1	-1,02
EPB41L4A-AS	-1,03
RPS26P11	-1,05
COG6	-1,05
SAFB	-1,07
LOC10065299	-1,07
BCR	-1,07
NCKIPSD	-1,08
OGT	-1,08
DNAAF1	-1,08
CDC73	-1,08
LDLRAD1	-1,1
WDR78	-1,1
SMPD3	-1,11
DHX38	-1,11

(II) malignant versus benign stroma

HGNC_symbol	logFC
CXCR7	3,49
EFEMP1	3,36
LOC10013399	3,35
SLIT2	3,28
CD53	2,86
FYB	2,85
NNMT	2,65
MAFB	2,49
EFEMP1	2,37
PLEK	2,35
ZDHHC14	2,29
ITGA4	2,21
CFB	2,15
RAPH1	2,11
S100A8	1,8
DNMT3B	1,78
ARL4C	1,76
LMF2	1,74
UTRN	1,72
AGFG1	1,67
S100A4	1,66
HLA-E	1,62
SPRY1	1,54
ADAMTSL5	1,48
CCDC14	1,46
NPC2	1,45
EMB	1,44
CXCR4	1,44
PYCR2	1,43
DHX8	1,42
CXCR4	1,4
LOC10065280	1,4
CMPK2	1,39
CSH1	1,36
BSN	1,32
GUCY1A3	1,32
LOC400707	1,3
BMPR2	1,24
CRIPAK	1,22
CAMK1G	1,21
C6orf106	1,2
LAPTM5	1,17
TCF4	1,16
TBC1D24	1,16
SNX18	1,14
FCER1G	1,13
OXSR1	1,13
CUEDC1	1,11
CFB	1,1
C4orf46	1,09
TAGLN	1,09
SLCO4A1	1,07
TRIM71	1,07
MYL8P	1,06
NFKBIZ	1,06
FKBP8	1,06
SLC17A7	1,04
STK17B	1,01
FLJ27352	-1,01
HNRNPA1	-1,01
ZNF334	-1,03
NUDCD2	-1,05
PAIP2B	-1,06
LOC284023	-1,07
HNRNPA1	-1,08
NMD3	-1,08
TLR6	-1,09
RPS4X	-1,1
KLHL11	-1,11
ATPIF1	-1,11
SRSF3	-1,13
HOXD3	-1,14
MRPL9	-1,16
RGNEF	-1,22
IGLCOR22-1	-1,22
STK33	-1,24
TSPAN5	-1,27
CMYA5	-1,31
NUFIP1	-1,34
STRN	-1,34
COQ5	-1,35
C3orf38	-1,35
CUL4A	-1,37
LGR4	-1,37
SECISBP2L	-1,39
LOC440297	-1,41
ANKRD36C	-1,43
ACTR8	-1,44
SUSD5	-1,44
METTL8	-1,45

(III) malignant versus benign tumors combined

HGNC_symbol	logFC
CENPF	2,56
IQGAP3	2,47
CXCR7	2,45
MX1	2,36
EPSTI1	2,31
RGS2	2,29
SPP1	2,22
CXCR4	2,17
RRM2	2,11
S100A8	2,07
SOCS3	2,03
NANP	2,01
OAS2	1,98
CLSPN	1,98
LYZ	1,97
VCAN	1,93
MXD1	1,88
LOC100133991	1,84
MT1G	1,81
LEF1	1,8
ISG15	1,76
IFI44L	1,75
TINAGL1	1,75
ZDHHC14	1,73
SLIT2	1,73
FGF9	1,72
DDX42	1,71
FGF18	1,7
PLEK	1,69
BMP7	1,69
NFKBIZ	1,69
ARL4C	1,68
CENPF	1,68
IFI44L	1,67
NCOA7	1,65
RGS5	1,65
STAT1	1,63
VCAN	1,63
NPC2	1,62
UBE2C	1,62
AURKA	1,61
UGP2	1,58
TNFSF10	1,58
OLFML2B	1,58
THBD	1,58
VCAN	1,57
FUT2	1,56
DLGAP5	1,55
CLIC4	1,54
MYCL1	1,53
KDM5C	1,53
IFI44L	1,52
SDC3	1,52
CD53	1,51
ANGEL2	1,5
IFITM1	1,49
ACTR3	1,48
FYB	1,48
CFB	1,47
SOX11	1,47
NCOA7	1,46
DHDDS	1,46
PIM2	1,46
RIT1	1,46
CDC6	1,45
CCT3	1,44
EFEMP1	1,44
CXCL17	1,44
CFI	1,43
GPX1	1,42
GPRC5A	1,42
ZNF566	1,41
ST20	1,4
APOC1	1,4
LHX1	1,39
GADD45B	1,39
LUM	1,39
NFKBIA	1,38
TATDN1	1,38
LYZ	1,37
CENPF	1,37
LCP1	1,36
SLC44A3	1,36
MMP15	1,36
CDC5L	1,36
PRDM1	1,35
CRABP2	1,34
APLP1	1,34
FCER1G	1,34
SOX11	1,34

CEP63	-1,13
MXRA7	-1,13
AGBL4-IT1	-1,14
IST1	-1,14
SPEF2	-1,14
LOC150197	-1,15
KIAA1430	-1,16
ALG12	-1,16
STK33	-1,16
LOC10050730	-1,17
TCEAL8	-1,18
FECH	-1,18
FBXO21	-1,19
CBR4	-1,19
PDK3	-1,2
IGBP1	-1,2
METAP1D	-1,2
CLDN18	-1,21
PHAX	-1,22
PJA2	-1,22
FLJ44342	-1,23
ATG2B	-1,23
ATP13A3	-1,23
TF	-1,24
BEX4	-1,25
ITGB3	-1,26
C18orf25	-1,27
OGT	-1,29
ANK2	-1,29
CEP70	-1,31
PCCB	-1,32
SPATA6L	-1,34
VWA3A	-1,35
N4BP2L2	-1,35
HYDIN	-1,35
TCF25	-1,38
LOC10013000	-1,41
ADAM6	-1,45
FN1	-1,48
CLCN3	-1,52
JAM2	-1,58
NAP1L3	-1,58
LENG8	-1,59
METAP1D	-1,6
TTC18	-1,6
SCAMP1	-1,61
VPS53	-1,63
ABCE1	-1,68
MAF	-1,69
TAPT1	-1,7
NRP2	-1,74
MRPL10	-1,78
KCTD12	-1,79
MGLL	-1,85
GRAMD3	-1,86
PPWD1	-1,88
MYLK3	-1,89
CCDC11	-1,9
NAIP	-1,9
LYSMD3	-1,93
LOC284009	-1,94
BIVM	-1,96
PTGS2	-2,14
OGT	-2,17
NEXN	-2,17
CYP4V2	-2,18
MBP	-2,19
DNAAF1	-2,22
ZNF177	-2,24
RORA	-2,32
LOC10050694	-2,49
GOSR1	-2,62
DNALI1	-2,62
HSDL1	-2,65
NME5	-2,7
ESYT3	-2,8
AR	-2,82
RBM24	-3,12
OVGP1	-3,16

NXT2	-1,45
PWRN1	-1,46
FLJ30901	-1,48
NUDT11	-1,48
PDIA3	-1,49
ABI2	-1,49
DOK6	-1,5
NFE2L1	-1,52
SMS	-1,55
ACTR5	-1,55
SIGLEC11	-1,59
METTL21B	-1,62
C12orf35	-1,63
LINC00515	-1,64
TRA2B	-1,66
PEG10	-1,67
C2orf29	-1,68
DEFB109P1B	-1,69
OCLN	-1,69
DPF3	-1,69
KDM5B	-1,7
HS3ST1	-1,72
ZNF737	-1,72
ZZZ3	-1,73
LOC100507308	-1,73
SIN3A	-1,75
PDZD4	-1,75
CDK12	-1,75
TOMM22	-1,76
PTCD3	-1,76
RALGAPB	-1,76
CCNJL	-1,76
NLE1	-1,76
BEX5	-1,78
FICD	-1,78
LCLAT1	-1,79
BICD1	-1,79
DENND5B	-1,79
SCYL2	-1,84
MYO5C	-1,88
CDH1	-1,89
GLS2	-1,89
PRDX4	-1,89
CD160	-1,94
TAB3	-1,95
RBM15	-1,96
BICD1	-1,96
RLIM	-1,98
TMEM39A	-1,98
WDR77	-2,02
LRIG2	-2,03
NRCAM	-2,04
SIKE1	-2,05
IFT81	-2,07
IGSF11	-2,07
ASXL3	-2,07
HUWE1	-2,11
C21orf62	-2,11
SNHG1	-2,17
PKNOX1	-2,17
PTCD3	-2,18
TH1L	-2,19
TRIM33	-2,2
PWRN2	-2,2
C12orf35	-2,21
ATF5	-2,21
SEL1L	-2,22
LOC100506948	-2,23
MEIS3P1	-2,26
ANP32A	-2,27
C14orf118	-2,28
ZFAND6	-2,28
ANK2	-2,28
RUNDC1	-2,33
CCDC77	-2,34
DYNC1H1	-2,35
USP22	-2,36
WNK1	-2,38
TMEM184C	-2,38
HMGB3	-2,4
TDGF1P3	-2,44
ZNF385B	-2,54
CLCN3	-2,57
SP1	-2,58
ZNF75D	-2,58
TMBIM4	-2,6
RPRD1B	-2,61
FAM223A	-2,68
DCTN5	-2,69
C12orf51	-2,69
FLG-AS1	-2,73
FAM120C	-2,81
MEG3	-2,88
UBFD1	-2,98
SNORD116@	-3,05
BICD1	-3,09

C5orf22	1,33
DSE	1,33
RAPH1	1,32
IFI27	1,32
CLDN6	1,31
LDLR	1,31
ADAR	1,31
HINT3	1,31
BLZF1	1,31
GNAO1	1,3
TOR1AIP2	1,29
SPP1	1,29
IRF9	1,29
DDX60L	1,29
BIRC2	1,29
NQO1	1,28
RAB13	1,28
RPS7	1,27
RBM10	1,26
BHLHE40	1,26
IFI44	1,26
SLC6A8	1,25
CDCP1	1,25
RUNX2	1,25
MCM6	1,25
ICMT	1,25
PVRL4	1,25
ATN1	1,24
C1orf106	1,24
IL17D	1,24
NXPE3	1,24
GPRC5A	1,24
MAL	1,24
FAM32A	1,23
RNPEP	1,23
STAU1	1,23
CMPK2	1,22
NFIL3	1,22
RGS1	1,22
NNMT	1,22
MAFB	1,22
COIL	1,22
TIAF1	1,22
PIM1	1,21
RABGAP1L	1,21
HIST2H2AB	1,21
RUNX2	1,21
CAMK2D	1,21
KLF13	1,21
COL3A1	1,21
MARK3	1,2
PRRX1	1,2
ORC6	1,2
PSMD6-AS2	1,2
C1orf116	1,2
BMF	1,2
VPS13D	1,2
BHLHE40	1,19
NOTCH3	1,19
CKAP2L	1,19
H1F0	1,19
VCAN	1,19
FN1	1,19
NUSAP1	1,19
CTGF	1,19
CXCR4	1,18
GPRC5A	1,18
FUCA2	1,18
SLIT2	1,18
DYRK2	1,18
LRRC41	1,17
TSC22D2	1,17
FN1	1,17
GNL3L	1,16
CYP1B1	1,16
STK17B	1,16
OAS3	1,16
CYP1B1	1,16
UCA1	1,15
COL6A3	1,15
COL5A2	1,15
BCL2L1	1,14
H1F0	1,14
BCL11A	1,14
IFIH1	1,14
RHOU	1,14
GPRC5B	1,14
CXCR4	1,13
CFB	1,13
FAM129A	1,13
IFIT1	1,13
TIGIT	1,13
SERTAD2	1,13
ATP1B1	1,13
STXBP6	1,13
HIST1H3I	1,12

AP2B1	-3,13
MLXIP	-3,17
ACSM4	-3,31
CCNB1	-3,43
NOL9	-3,52
TSPAN5	-3,82

ANKRD27	1,12
PDLIM5	1,12
SFRP2	1,12
PRRX1	1,12
THBS1	1,12
RASGEF1B	1,11
ARL4C	1,11
FURIN	1,11
SOX17	1,11
PVRL3	1,11
IL1R1	1,11
LGALS1	1,11
FGF7	1,1
CDCA7	1,1
S100A4	1,09
LOC284581	1,09
IL1RAP	1,09
RRM2	1,09
EFEMP1	1,09
CCDC102B	1,09
VCAN	1,08
GCH1	1,08
UCA1	1,08
PRIMA1	1,08
ZDHHC9	1,07
FAM129A	1,07
CCBP2	1,07
PIGA	1,07
ARL6IP5	1,07
ICMT	1,07
SNX9	1,07
DCUN1D3	1,07
CXCL3	1,06
STK17B	1,06
EOGT	1,06
TFE3	1,06
WDR5B	1,06
FOXQ1	1,06
UNC5C	1,06
LOC100653329	1,06
MAN1A1	1,06
STXBP6	1,06
COL3A1	1,06
NAMPT	1,05
CAMK2D	1,05
C19orf55	1,05
ILDR2	1,05
ITGA4	1,05
DTX3L	1,05
STAT1	1,04
HLA-L	1,04
TRNT1	1,04
FAM174B	1,04
KIAA1919	1,04
SFRP2	1,04
DNMT3B	1,03
RAB3D	1,03
NR2F6	1,03
LDLR	1,03
CDC42EP4	1,03
BMPR2	1,02
LRP2	1,02
EMP1	1,02
DSC3	1,02
HIST1H4L	1,02
IL1RN	1,02
ERC1	1,02
CSNK2A1P	1,02
PHF20	1,02
IFI44L	1,01
APOLD1	1,01
RNF19A	1,01
CBX4	1,01
KCNQ1OT1	1,01
CCDC109B	1
TAGLN2	1
GPRC5A	1
LOC100652809	1
KIRREL	1
RAPH1	1
HMGCS2	1
HOXD4	-1
TMEM231	-1
ZNF286A	-1
FEM1B	-1
PAK2	-1
NFE2L1	-1
SCD5	-1
KLHL4	-1,01
DTWD1	-1,01
RPRD1A	-1,01
RAB2B	-1,01
CCDC146	-1,01
BPTF	-1,01
PAFAH1B1	-1,01
H19	-1,01

GNAS	-1,01
PCOLCE2	-1,01
MAGED1	-1,01
USP32P2	-1,01
GNG2	-1,01
TDRD3	-1,01
NTS	-1,01
FGFR1OP2	-1,01
NRP2	-1,01
CSRNP3	-1,01
IGBP1	-1,02
KIAA1430	-1,02
SRSF12	-1,02
DNAJC3	-1,02
UBE3B	-1,02
MIB1	-1,02
PAIP1	-1,02
TCFL5	-1,02
ACAP2	-1,02
VAMP4	-1,02
IQGAP1	-1,02
RASA1	-1,02
ENC1	-1,02
MORC4	-1,02
WNK1	-1,02
EPG5	-1,02
THUMPD1	-1,02
SLC4A4	-1,02
CDH1	-1,02
SPG20	-1,02
DPF3	-1,03
SMAD2	-1,03
NFE2L1	-1,03
LAMP2	-1,03
ZNF615	-1,03
SRRM1	-1,03
RHOXF2	-1,03
ANO4	-1,03
ZNF420	-1,03
C5orf15	-1,03
SLC35B1	-1,03
NEFH	-1,03
AKAP12	-1,03
TBL1X	-1,03
PWRN1	-1,04
TF	-1,04
LEPR	-1,04
UBXN7	-1,04
ZNF641	-1,04
NID2	-1,04
DDX17	-1,04
NRK	-1,04
SPRR2E	-1,04
BICD1	-1,04
RPS23	-1,04
CDK12	-1,04
GOLGA8A	-1,04
CDH2	-1,04
BRWD1	-1,04
NAA30	-1,04
FAM165B	-1,04
LRAT	-1,04
MEG3	-1,04
NFE2L1	-1,05
KDM5B	-1,05
EML5	-1,05
IFT57	-1,05
EPM2AIP1	-1,05
ESYT3	-1,05
ABCA10	-1,05
LEPR	-1,05
MIER1	-1,05
ADAMTS3	-1,05
GRB14	-1,05
KLHL5	-1,05
METAP1D	-1,06
CDK12	-1,06
LARS2	-1,06
LOC100507031	-1,06
GPD2	-1,06
ETV5	-1,06
CNBP	-1,06
N4BP2L1	-1,06
RBM25	-1,06
NLRP1	-1,06
OSBPL1A	-1,06
B3GALNT2	-1,06
CEP63	-1,07
SMAD2	-1,07
THOC2	-1,07
SRSF11	-1,07
U2SURP	-1,07
KCTD12	-1,07
KLC4	-1,07
C14orf118	-1,07
PGAM5	-1,07

TTC8	-1,07
ZMYM2	-1,07
LOC10013056(	-1,07
JPX	-1,08
C1orf168	-1,08
ESD	-1,08
SPRR2D	-1,08
SCN2A	-1,08
KLF3	-1,08
C21orf62	-1,08
ZFP90	-1,08
BOD1L1	-1,08
C4orf29	-1,08
LOC10050696(	-1,09
TAF7	-1,09
MYO5C	-1,09
TMCC1	-1,09
THEM4	-1,09
PDHB	-1,09
ZNF207	-1,09
SRSF1	-1,09
PRPF39	-1,09
STX8	-1,09
TMOD2	-1,09
ANKRD29	-1,09
TCF12	-1,09
CPD	-1,09
LOC10028923(	-1,09
C7	-1,09
C12orf35	-1,1
DEFB109P1B	-1,1
OCLN	-1,1
MTND5P2	-1,1
RALBP1	-1,1
RBM26	-1,1
UBE2A	-1,1
SEPP1	-1,1
DCLK1	-1,1
TRIM2	-1,1
TNRC6B	-1,1
MBOAT2	-1,1
KAT2B	-1,1
ARHGAP24	-1,1
NAV3	-1,1
LINC00515	-1,11
SCN7A	-1,11
PDZD4	-1,11
TSPAN6	-1,11
LTB4R	-1,12
CYB5D1	-1,12
SLITRK4	-1,12
SCFD1	-1,12
MINA	-1,12
USMG5P1	-1,12
GPX3	-1,12
PRTG	-1,12
ANKRD36C	-1,13
C1orf173	-1,13
IGSF11	-1,13
TAB3	-1,13
ITGA6	-1,13
ADAMTS9-AS1	-1,13
RBBP6	-1,13
B4GALT4	-1,13
SPAG8	-1,13
ZNF14	-1,13
PPP2R2C	-1,13
NDEL1	-1,13
PTPLB	-1,13
PKHD1L1	-1,13
KLHL11	-1,13
NRP2	-1,13
TGFB2	-1,13
DENND5B	-1,14
SOBP	-1,14
UNC119B	-1,14
URI1	-1,14
GTF2F1	-1,14
ALDH3A2	-1,14
REV1	-1,14
FAT4	-1,14
WNT5A	-1,14
SYNJ2BP	-1,14
BEX4	-1,15
SPATA18	-1,15
PNN	-1,15
FAM210B	-1,15
ZNF552	-1,15
LOC10012910(	-1,15
PDCD7	-1,15
DERL2	-1,15
MINOS1	-1,15
HNRNPA1	-1,15
PEBP1	-1,16
SERPINE2	-1,16
ZNF407	-1,16

NRIP1	-1,16
TMEM39A	-1,16
MARK1	-1,16
LDOC1	-1,16
HELZ	-1,16
ZNF318	-1,16
CPSF6	-1,17
TAS2R46	-1,17
FAM18B1	-1,17
MED28	-1,17
LOC100289627	-1,17
PHKB	-1,17
TBC1D4	-1,17
DYNC2LI1	-1,18
JAM2	-1,18
FAM216B	-1,18
F8	-1,18
PPWD1	-1,18
PCTP	-1,18
PDXDC2P	-1,18
WNK3	-1,18
SPATA7	-1,18
BCYRN1	-1,18
STK33	-1,19
PAPD4	-1,19
HERC2P3	-1,19
GMFB	-1,19
SMAD4	-1,19
PPIAP21	-1,19
SEL1L	-1,19
SNHG5	-1,19
EPS15	-1,19
MBOAT1	-1,19
NDFIP1	-1,19
CYTH2	-1,19
RSPH1	-1,19
FBXW8	-1,19
PURA	-1,19
ZNF264	-1,2
ABI2	-1,2
LOC100507547	-1,2
CLCN3	-1,2
ZNF493	-1,2
PPP2R5C	-1,2
GLG1	-1,2
PCYOX1L	-1,2
CYSTM1	-1,2
RPL7	-1,2
RAP1GAP2	-1,2
UTP20	-1,2
SCGB1D2	-1,2
DOK6	-1,21
METTL21B	-1,21
IFT81	-1,21
C18orf25	-1,21
ANK2	-1,21
KCTD12	-1,21
APH1B	-1,21
ZNF681	-1,21
RBM26	-1,21
UBFD1	-1,21
ERO1LB	-1,21
ZZZ3	-1,22
ABHD10	-1,22
PWRN2	-1,22
GSTA3	-1,22
SNX18	-1,22
C14orf135	-1,22
RPS26	-1,22
KLC1	-1,22
N4BP2L1	-1,22
LUC7L3	-1,22
ROBO2	-1,22
PTCD3	-1,23
NOTCH2NL	-1,23
MUC16	-1,23
GOLGA6L5	-1,24
RASA1	-1,24
AHCYL1	-1,24
WDR19	-1,24
RMST	-1,24
TBP	-1,24
CD302	-1,24
KLF11	-1,24
DHX40	-1,24
SHISA2	-1,24
PHEX	-1,25
ADGB	-1,25
XPNPEP3	-1,25
LONRF2	-1,25
ESCO1	-1,25
STAG2	-1,25
SHROOM3	-1,25
NEXN	-1,25
N4BP2L2	-1,26
RBM15	-1,26

Feb 03	-1,26
GDNF	-1,26
LRIG2	-1,26
RBPJ	-1,26
TAS2R50	-1,26
RASA1	-1,26
CDKN2AIP	-1,26
TCTN2	-1,26
ACSS3	-1,26
SLC25A27	-1,26
RBM24	-1,26
RUFY3	-1,27
FAM98A	-1,27
TERF2IP	-1,28
TH1L	-1,28
DCAF16	-1,28
IPW	-1,28
EGLN3	-1,28
HDHD2	-1,28
ARMCX4	-1,28
ZNF84	-1,29
NDEL1	-1,29
NAP1L2	-1,29
GNAS	-1,29
KIF5C	-1,29
PNRC2	-1,29
RAN	-1,3
WDR77	-1,3
FAM227A	-1,3
EPB41L2	-1,3
APOOL	-1,3
DNAH12	-1,3
SCN7A	-1,3
RAD52	-1,3
SYNPO2	-1,3
CCDC11	-1,31
MEIS3P1	-1,31
GMNC	-1,31
PTGS2	-1,31
FAM120C	-1,31
ATP1A1	-1,32
RFFL	-1,32
ZNF776	-1,32
WNT5A	-1,32
SERPINE2	-1,32
C5orf43	-1,32
VPS53	-1,33
GPATCH8	-1,33
SCD5	-1,33
NOTCH2	-1,33
RLIM	-1,33
FZD7	-1,33
TMEM184C	-1,34
DNALI1	-1,34
RPRD1B	-1,34
KCTD12	-1,34
SRSF3	-1,34
CRYZ	-1,34
PGM1	-1,34
OR7E47P	-1,34
PPM1K	-1,35
MBLAC2	-1,35
SPRYD7	-1,35
C16orf52	-1,35
HDHD2	-1,35
MBNL2	-1,35
CHRFAM7A	-1,35
TCEAL2	-1,35
GREB1	-1,36
THUMPD1	-1,36
C17orf57	-1,36
CSRNP3	-1,36
AMY2B	-1,36
NLE1	-1,37
SNX1	-1,37
GUSBP4	-1,37
C16orf80	-1,37
C12orf51	-1,37
MRPL40	-1,37
MSI2	-1,37
ATP9A	-1,37
ZMAT1	-1,38
ZNF711	-1,38
ANAPC4	-1,38
TDGF1P3	-1,38
NOB1	-1,38
SPATA17	-1,38
RBMS1	-1,38
NAP1L3	-1,38
RPRM	-1,38
DNAAF1	-1,39
PHKB	-1,39
TM4SF19	-1,39
TTC3	-1,4
FAM13A	-1,4
LAMB3	-1,4

RPS8	-1,4
RUNDC1	-1,4
TPP2	-1,41
BAZ2B	-1,41
ANK2	-1,41
MTHFSD	-1,41
WDR96	-1,41
EPB41L2	-1,41
CPD	-1,41
CCDC144B	-1,41
PIAS2	-1,42
RBMX2	-1,42
TMEM170A	-1,42
SERPINB8	-1,43
CCNB1	-1,43
BEX5	-1,44
ACTR5	-1,44
LRRC49	-1,44
FAM82A2	-1,44
ARSG	-1,44
LOC10027222	-1,45
C12orf35	-1,46
UBXN8	-1,46
LPGAT1	-1,46
ASXL3	-1,46
HOXD4	-1,46
PPM1L	-1,46
MLH3	-1,47
UBE2I	-1,47
LOC10050730	-1,48
SLC40A1	-1,48
ECHDC2	-1,48
ID4	-1,49
PHACTR3	-1,49
NAV3	-1,49
DZIP3	-1,5
GMNC	-1,5
SYTL4	-1,5
ARMCX4	-1,5
PCDHB5	-1,5
FLRT2	-1,5
HSDL1	-1,51
PROM1	-1,51
GOSR1	-1,52
BICD1	-1,52
PGGT1B	-1,52
ZNF75D	-1,52
ARHGAP29	-1,52
SNAP91	-1,52
CEP70	-1,53
ZNF385B	-1,53
WHAMM	-1,53
HAUS3	-1,53
ZFX	-1,53
PCMTD2	-1,53
EIF3C	-1,53
C1orf63	-1,54
DMD	-1,54
RPL15	-1,55
SCAMP1	-1,55
ZFAND6	-1,55
TRPC1	-1,55
SLC40A1	-1,55
UNC80	-1,55
CILP	-1,55
MEG3	-1,56
FAT1	-1,56
PWRN2	-1,57
CLCN3	-1,57
RORA	-1,57
RASGRF2	-1,58
SDK2	-1,59
PLCXD3	-1,59
WASF3	-1,59
CYP4V2	-1,6
ZNF204P	-1,6
LUC7L	-1,61
C3orf15	-1,61
LOC10050696	-1,62
MBD1	-1,62
MRPL10	-1,63
NOTCH2NL	-1,63
SLC5A3	-1,63
MTX3	-1,63
RPRD1B	-1,64
TMEM97	-1,64
PAPD4	-1,65
ZNF177	-1,65
PIK3C3	-1,65
EDA2R	-1,66
BCL2	-1,66
TXNDC16	-1,66
ATXN1	-1,67
SERPINE2	-1,68
FOXO1	-1,68
EDIL3	-1,68

GRAMD3	-1,69
MYLK3	-1,69
ZXDC	-1,69
DSEL	-1,69
NLGN4X	-1,69
SP1	-1,7
TMEM170A	-1,7
KAT2B	-1,7
FAM172A	-1,71
NOL9	-1,71
WNT5A	-1,72
ARRDC4	-1,72
PEG3	-1,72
OGT	-1,73
CHRFAM7A	-1,73
ACSM4	-1,74
ZMAT1	-1,74
SLC40A1	-1,76
NME5	-1,76
ID4	-1,77
PEG3	-1,78
PRNP	-1,78
GMNC	-1,78
ZMAT1	-1,79
TAPT1	-1,8
GPRASP1	-1,8
PEG3	-1,81
FLG-AS1	-1,82
ZNF207	-1,83
RSPH1	-1,84
COPS8	-1,85
STX2	-1,87
PEG3	-1,88
TCF21	-1,88
TMEM161B-AS	-1,89
ATRNL1	-1,89
EP400	-1,9
GATM	-1,91
C1orf168	-1,92
TMBIM4	-1,93
MBTPS1	-1,93
AKAP10	-1,93
FTX	-1,94
TSPAN8	-1,94
PLCXD3	-1,97
PEG3	-1,97
LOC10050694	-1,99
MEG3	-2
BICD1	-2,01
OVGP1	-2,03
NLK	-2,03
SPRR2F	-2,1
BIVM	-2,11
AR	-2,12
F8	-2,14
MST1P9	-2,14
PHEX	-2,15
CD22	-2,17
TSHZ1	-2,2
LOC284244	-2,23
BRWD3	-2,25
C1orf173	-2,26
SNORD116@	-2,31
LOC10050694	-2,42
RPL37	-2,54
MLXIP	-2,61
TPRG1	-2,75
TSPAN5	-2,76
