# Supplementary material for: Discovery and Validation of Novel Biomarkers for Detection of Epithelial Ovarian Cancer
Source: Cells. 2019 Jul 12;8(7):713. doi: 10.3390/cells8070713 (PMC6678810; doi:10.3390/cells8070713)
Supplement: Supplementary file 1 [file cells-08-00713-s001.zip › Suppl. Table 3.pdf]

Table S3. 152 gene signature

HGNC_symb	logFC	fold change
EFEMP1	3,36	10,27
SLIT2	3,28	9,71
RGS2	3,01	8,06
DTL	2,92	7,57
CD53	2,86	7,26
MMP15	2,69	6,45
ICMT	2,52	5,74
SPP1	2,5	5,66
AURKA	2,46	5,50
PLEK	2,35	5,10
ZDHHC14	2,29	4,89
ITGA4	2,21	4,63
ISG15	2,2	4,59
CFB	2,15	4,44
RAPH1	2,11	4,32
S100A8	2,07	4,20
NANP	2,01	4,03
OAS2	1,98	3,94
VCAN	1,93	3,81
CLDN6	1,86	3,63
LRP2	1,86	3,63
NPC2	1,85	3,61
RALBP1	1,81	3,51
CHCHD3	1,8	3,48
ARL4C	1,76	3,39
TINAGL1	1,75	3,36
LMF2	1,74	3,34
FGF9	1,72	3,29
UTRN	1,72	3,29
FGF18	1,7	3,25
BMP7	1,69	3,23
RGS5	1,65	3,14
OLFML2B	1,58	2,99
THBD	1,58	2,99
TNFSF10	1,58	2,99
FUT2	1,56	2,95
CLIC4	1,54	2,91
SPRY1	1,54	2,91
KDM5C	1,53	2,89
CALR	1,52	2,87
SDC3	1,52	2,87
UBE2D3	1,51	2,85
IFITM1	1,49	2,81
ADAMTSL5	1,48	2,79
NOTCH3	1,47	2,77
PTP4A2	1,47	2,77
RIT1	1,46	2,75

MELK	1,45	2,73
CCT3	1,44	2,71
CXCL17	1,44	2,71
EMB	1,44	2,71
CFI	1,43	2,69
RHBDL2	1,43	2,69
ZFYVE16	1,41	2,66
APOC1	1,4	2,64
LUM	1,39	2,62
NFKBIA	1,38	2,60
LYZ	1,37	2,58
CSH1	1,36	2,57
LCP1	1,36	2,57
SLC44A3	1,36	2,57
APLP1	1,34	2,53
FCER1G	1,34	2,53
DSE	1,33	2,51
IFI27	1,32	2,50
LDLR	1,31	2,48
GNAO1	1,3	2,46
TOR1AIP2	1,29	2,45
NQO1	1,28	2,43
RAB13	1,28	2,43
SNX5	1,27	2,41
CDCP1	1,25	2,38
PVRL4	1,25	2,38
SLC6A8	1,25	2,38
GPRC5A	1,24	2,36
IL17D	1,24	2,36
MAL	1,24	2,36
RNPEP	1,24	2,36
CRIPAK	1,23	2,35
RGS1	1,22	2,33
CAMK1G	1,22	2,33
COL3A1	1,21	2,31
PIM1	1,21	2,31
BMF	1,21	2,31
C1orf116	1,2	2,30
C6orf106	1,2	2,30
MARK3	1,2	2,30
PRRX1	1,2	2,30
CTGF	1,2	2,30
FN1	1,19	2,28
SCD	1,19	2,28
FUCA2	1,19	2,28
LAPTM5	1,18	2,27
LRRC41	1,17	2,25
VKORC1L1	1,17	2,25
CYP1B1	1,17	2,25
COL5A2	1,16	2,23

COL6A3	1,15	2,22
BCL2L1	1,15	2,22
GPRC5B	1,14	2,20
IFIH1	1,14	2,20
RHOU	1,14	2,20
SNX18	1,14	2,20
ATP1B1	1,14	2,20
FAM129A	1,13	2,19
STXBP6	1,13	2,19
TIGIT	1,13	2,19
PDLIM5	1,13	2,19
SFRP2	1,12	2,17
THBS1	1,12	2,17
FURIN	1,12	2,17
IL1R1	1,11	2,16
LGALS1	1,11	2,16
PVRL3	1,11	2,16
FGF7	1,11	2,16
IL1RAP	1,1	2,14
SEMA4C	1,09	2,13
TAGLN	1,09	2,13
PRIMA1	1,09	2,13
AP2M1	1,08	2,11
ARL6IP5	1,07	2,10
CCBP2	1,07	2,10
PIGA	1,07	2,10
SLCO4A1	1,07	2,10
SNX9	1,07	2,10
ZDHHC9	1,07	2,10
ATP6V0A2	1,07	2,10
CXCL3	1,06	2,08
FKBP8	1,06	2,08
MAN1A1	1,06	2,08
RPN2	1,06	2,08
UNC5C	1,06	2,08
WDR5B	1,06	2,08
CAMK2D	1,06	2,08
RALY	1,05	2,07
FAM174B	1,05	2,07
KIAA1919	1,04	2,06
SLC17A7	1,04	2,06
CDC42EP4	1,04	2,06
NR2F6	1,03	2,04
RAB3D	1,03	2,04
BMPR2	1,03	2,04
DSC3	1,02	2,03
EMP1	1,02	2,03
ERC1	1,02	2,03
HIST1H4L	1,02	2,03
IL1RN	1,02	2,03

APOLD1	1,02	2,03
RNF19A	1,01	2,01
CCDC109B	1,01	2,01
KIRREL	1	2,00
TAGLN2	1	2,00
