# Supplementary material for: Discovery and Validation of Novel Biomarkers for Detection of Epithelial Ovarian Cancer
Source: Cells. 2019 Jul 12;8(7):713. doi: 10.3390/cells8070713 (PMC6678810; doi:10.3390/cells8070713)
Supplement: Supplementary file 1 [file cells-08-00713-s001.zip › Suppl. Table 4.pdf]

Table S4A: Tissue based results are shown

Model	Full	VCAN	NR2F6	MELK	ISG15	MMP15	CLDN6	DTL	MAL	CFB	AURKA	BMP7	FUT2	FGF18	Average
TP	23	14	21	20	20	21	22	21	20	21	20	18	20	19	20,25
FP		9	2	3	3	2	1	2	3	2	3	5	3	4	2,75
Sensitivity	100	82	91	100	82	100	100	100	73	91	82	64	82	91	88
Specificity	100	42	92	75	92	83	92	83	100	92	92	92	92	75	88
PPV	100	56	91	79	90	85	92	85	100	91	90	88	90	77	88

The prediction performance of the twelve tissue nanostring validated biomarker candidates, a model based on all twelve and the average performance of the twelve markers along with VCAN is shown. Sensitivity ranged from 20 to 90% with an average of 68% while specificity ranged from 30% to 100% with average of 58%. The PPV ranged from 53% to 100% and averaged at 66%. Malignant has been set as default class i.e. had to be predicted and a sensitivity of 50% states that 50% of all malignant samples have been correctly identified

Table S4B: Blood based results are shown

Model	Full	VCAN	NR2F6	MELK	ISG15	MMP15	CLDN6	DTL	MAL	CFB	AURKA	BMP7	FUT2	FGF18	Average
TP	18	14	14	14	14	13	13	13	12	12	12	11	11	11	12,5
FP	4	6	6	6	6	7	7	7	8	8	8	9	9	9	7,5
Sensitivity	90	60	90	70	80	60	40	70	80	90	20	50	90	70	68
Specificity	70	80	50	70	60	70	90	60	40	30	100	60	20	40	58
PPV	75	75	64	70	67	67	80	64	57	56	100	56	53	54	66

The prediction performance of the twelve blood nanostring validated biomarker candidates, a model based on all twelve and the average performance of the twelve markers along with VCAN is shown. Sensitivity ranged from 20 to 90% with an average of 68% while specificity ranged from 30% to 100% with average of 58%. The PPV ranged from 53% to 100% and averaged at 66%

Legend

TP = True Positives
FP = False Positives
Sensitivity = True Positive Rate
Specificity = True Negative Rate
PPV = Positive Predictive Value
