# Supplementary material for: Discovery and Validation of Novel Biomarkers for Detection of Epithelial Ovarian Cancer
Source: Cells. 2019 Jul 12;8(7):713. doi: 10.3390/cells8070713 (PMC6678810; doi:10.3390/cells8070713)
Supplement: Supplementary file 1 [file cells-08-00713-s001.zip › Supplementary Figure S1.pdf]

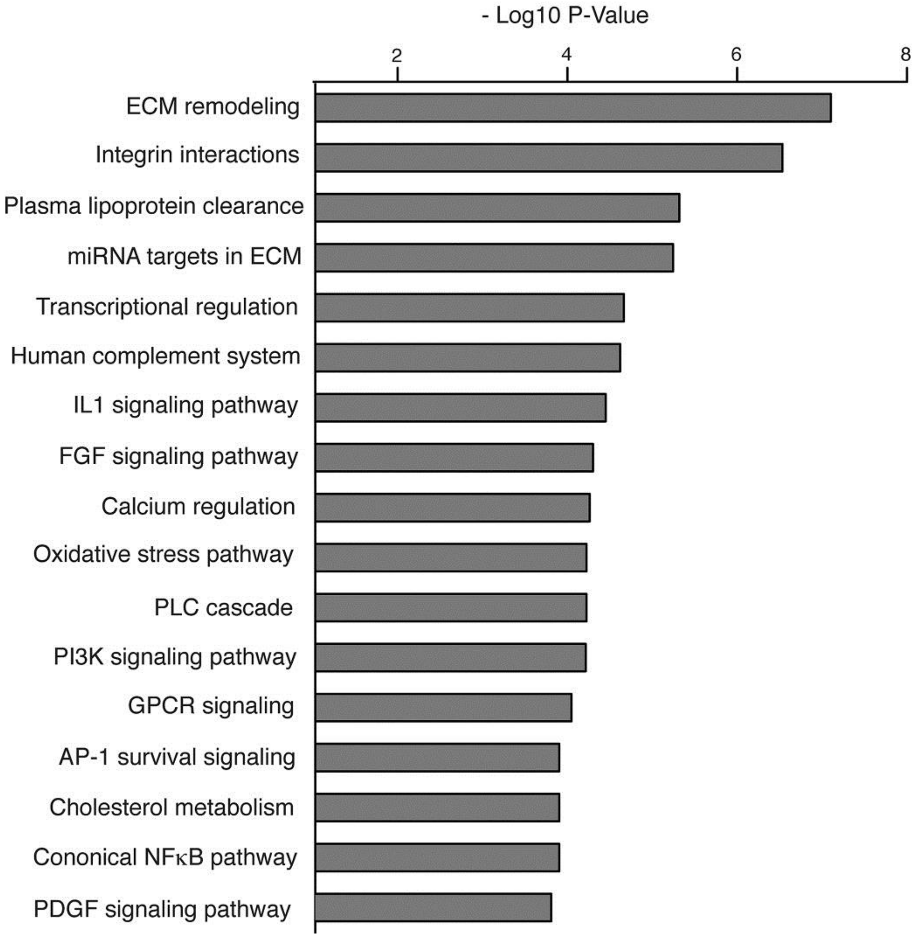

Supplementary Figure S1: Gene-set over-representation analysis of candidate biomarkers. The biological processes that the 152 candidates were associated with were uncovered by gene-set over-representation analysis (GSOA) using the ConsensusPath database (http://cpdb.molgene.mpg.de) The 17 significantly over-represented processes and pathways with greatest statistical power are depicted (P-value < 0.002).
