# Supplementary material for: Discovery and Validation of Novel Biomarkers for Detection of Epithelial Ovarian Cancer
Source: Cells. 2019 Jul 12;8(7):713. doi: 10.3390/cells8070713 (PMC6678810; doi:10.3390/cells8070713)
Supplement: Supplementary file 1 [file cells-08-00713-s001.zip › Supplementary Figure S2.pdf]

A

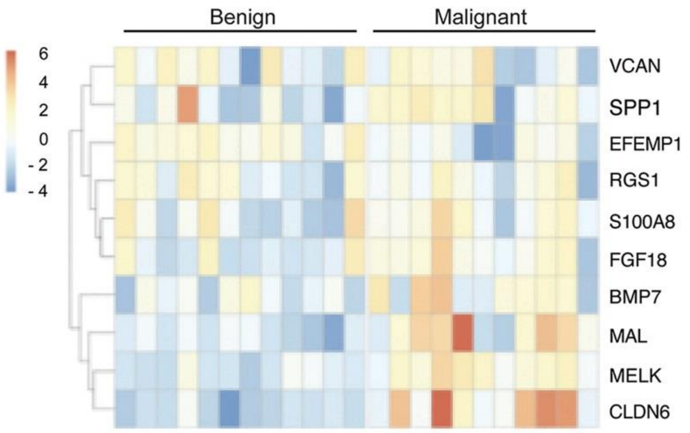

B

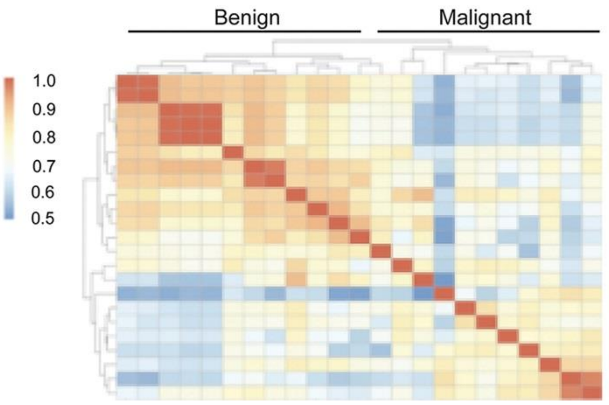

Supplementary Figure S2: Expression-levels of candidate biomarkers and pair-wise correlation of underlying samples. Plot A shows the absolute expression of candidate biomarkers for each sample. Plot B shows the correlation of the samples to identify outliers or incoherent clustering. The expression-correlation of the samples shows a clustering according to malignant and benign subtype, respectively, however, the expression levels of the biomarker candidates between and within groups vary and show considerable heterogeneity as shown in Plot A.
