# Supplementary material for: Discovery and Validation of Novel Biomarkers for Detection of Epithelial Ovarian Cancer
Source: Cells. 2019 Jul 12;8(7):713. doi: 10.3390/cells8070713 (PMC6678810; doi:10.3390/cells8070713)
Supplement: Supplementary file 1 [file cells-08-00713-s001.zip › Supplementary Figure S3.pdf]

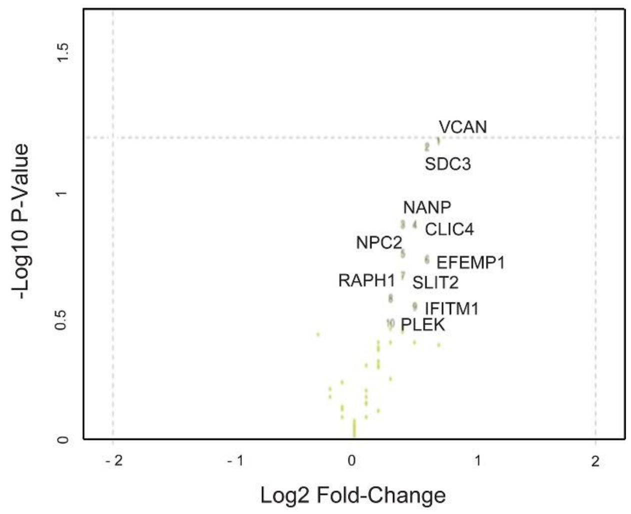

Supplementary Figure S3: Volcano plot of malignant versus benign gene expression in PAXgene samples from blood. NanoString-measured Log-Fold-Changes and associated P-values for differential expression of candidate biomarkers between benign (negative values) and in malignant tissue (positive values) are shown. VCAN (P-value 0.052) and SDC3 (P-value 0.055) show the lowest P-values but are not significantly differentially expressed. VCAN and SDC3 are however, significant for differential expression and show a comparable Log-Fold change based on data from the CSIOVDB database shown in Figure 4.
