# Supplementary material for: Discovery and Validation of Novel Biomarkers for Detection of Epithelial Ovarian Cancer
Source: Cells. 2019 Jul 12;8(7):713. doi: 10.3390/cells8070713 (PMC6678810; doi:10.3390/cells8070713)
Supplement: Supplementary file 1 [file cells-08-00713-s001.zip › Supplementary Figure S5.pdf]

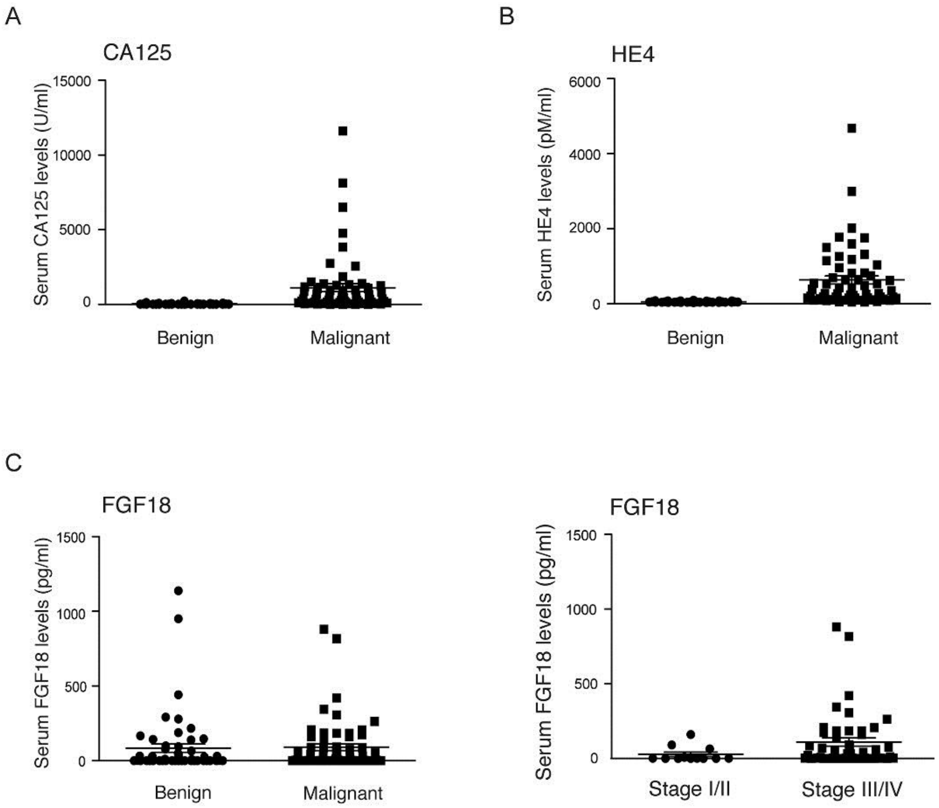

Supplementary Figure S5. Protein expression levels of biomarkers in serum. Levels of A) CA125, B) HE4 and C) FGF18 were determined in serum of patients with benign gynecological conditions (N=56) and ovarian cancer (N=60). All proteins were quantified using specific ELISA assays.
